# In-silico and in-vitro study of novel antimicrobial peptide AM1 from *Aegle marmelos* against drug-resistant *Staphylococcus aureus*

**DOI:** 10.1038/s41598-024-76553-0

**Published:** 2024-10-28

**Authors:** Rudra Awdhesh Kumar Mishra, Gothandam Kodiveri Muthukaliannan

**Affiliations:** School of Biosciences and Technology, Vellore Institute of Technology, Vellore, 632014 Tamil Nadu India

**Keywords:** *Aegle marmelos*, Antimicrobial peptides, DBAASP server, Drug-resistant *Staphylococcus aureus*, MMPBSA, Computational biology and bioinformatics, Microbiology

## Abstract

Antimicrobial peptides have garnered increasing attention as potential alternatives due to their broad-spectrum antimicrobial activity and low propensity for developing resistance. This is for the first time; proteome sequences of *Aegle marmelos* were subjected to in-silico digestion and AMP prediction were performed using DBAASP server. After screening the peptides on the basis of different physiochemical property, peptide sequence GKEAATKAIKEWGQPKSKITH (AM1) shows the maximum binding affinity with − 10.2 Kcal/mol in comparison with the standard drug (Trimethoprim) with − 7.4 kcal/mol and − 6.8 Kcal/mol for DHFR and SaTrmK enzyme respectively. Molecular dynamics simulation performed for 300ns, it has been found that peptide was able to stabilize the protein more effectively, analysed by RMSD, RMSF, and other statistical analysis. Free binding energy for DHFR and SaTrmK interaction from MMPBSA analysis with peptide was found to be -47.69 and − 44.32 Kcal/mol and for Trimethoprim to be -13.85 Kcal/mol and − 11.67 Kcal/mol respectively. Further in-vitro study was performed against Methicillin Susceptible *Staphylococcus aureus* (MSSA), Methicillin Resistant *Staphylococcus aureus* (MRSA), Multi-Drug Resistant *Staphylococcus aureus* (MDR-SA) strain, where MIC values found to be 2, 4, and 8.5 µg/ml lesser in comparison to trimethoprim which has higher MIC values 2.5, 5, and 9.5 µg/ml respectively. Thus, our study provides the insight for the further in-vivo study of the peptides against multi-drug resistant *S. aureus*.

**Supplementary Information:**

The online version contains supplementary material available at 10.1038/s41598-024-76553-0.

## Introduction

In the realm of infectious diseases, *Staphylococcus aureus* has long posed a formidable challenge to healthcare system worldwide^1^. This bacterium’s ability to adapt, evolve, and develop resistance mechanisms has rendered it a leading cause of both hospital-acquired and community-associated infections^2,3^. Complications arising from *S. aureus* infections range from mild skin and soft tissue infections to threatening conditions such as pneumonia, endocarditis and sepsis^4^. The emergence and spread of antibiotic resistance strains, particularly methicillin-resistant *S. aureus* (MRSA) have further heightened the urgency for alternative treatment strategies. Among those alternatives, antimicrobial peptides have garnered significant attention for their potent antimicrobial properties and unique mechanism of action^5^.

Unlike traditional antibiotics that target specific cellular components or metabolic pathways, AMPs possess a broad range of targets, including the bacterial cell membrane, intracellular components, and even DNA/RNA. AMPs interact with the bacterial membrane causing pore formation and permeabilization, leading to the leakage of cellular contents and ultimately bacterial death^6,7^. This mode of action makes it challenging for bacteria to develop resistance, as it would require significant alterations in their fundamental structure and function. Further understanding of AMPs and the development of effective delivery systems may pave the way for innovative therapies that could revolutionize the treatment of *Staphylococcus aureus* infections and help mitigate the threat of antibiotic resistance^8^.

The emergence of antibiotic-resistant strains like MRSA and MDR-SA has complicated treatment. Initially, beta-lactam antibiotics such as methicillin were effective by targeting penicillin-binding proteins essential for cell wall synthesis. However, MRSA developed through the mecA gene encoding PBP2a, which has low affinity for beta-lactams. Vancomycin became the next line of defense but overuse led to vancomycin-intermediate and -resistant strains (VISA and VRSA)^5^. Linezolid and daptomycin were introduced to inhibit protein synthesis and disrupt cell membranes, respectively, but resistance emerged due to genetic mutations and membrane alterations. Tetracyclines and Rifampin have been used in combination therapies but face increasing resistance. These challenges highlight the urgent need for new therapeutics^4,6^.

Two critical enzymes that contribute to the survival and pathogenicity of *S. aureus* are Dihydrofolate Reductase (DHFR) and 1A22-tRNA Methyltransferase (SaTrmK). *Staphylococcus aureus* relies on the efficient functioning of various enzymes to maintain its survival, growth, and virulence during infection. Two such critical enzymes are Dihydrofolate Reductase (DHFR) and 1A22-tRNA Methyltransferase (SaTrmK). The suppression of these enzymes has profound consequences on the bacterial cell, which can ultimately lead to its death. DHFR is a key enzyme in the folate biosynthesis pathway, which is essential for the production of nucleotides—the building blocks of DNA and RNA. Specifically, DHFR catalyzes the reduction of dihydrofolate to tetrahydrofolate, a necessary cofactor for the synthesis of thymidylate (a precursor of thymine) and purines (adenine and guanine). These nucleotides are required for DNA replication and repair, as well as RNA transcription. Suppressing DHFR will leads to a depletion of tetrahydrofolate, which is crucial for the synthesis of thymidylate and purines. As a result, the cell’s ability to synthesize DNA is severely compromised, leading to stalled DNA replication and an accumulation of DNA damage. The disruption of DNA synthesis triggers a checkpoint response, leading to cell cycle arrest. In *Staphylococcus aureus*, this means the bacterium can no longer progress through the cell cycle, halting its proliferation and growth. The inability to replicate DNA and repair damage eventually leads to cell death, as the bacterium cannot sustain its necessary biological processes. In the context of infection, this would result in the reduction of bacterial load and the alleviation of symptoms in the host^45^. Whereas, SaTrmK is involved in the post-transcriptional modification of transfer RNA (tRNA), specifically through the methylation of adenine residues. This modification is essential for stabilizing tRNA and ensuring accurate decoding of mRNA during protein synthesis. Protein synthesis is a fundamental process for bacterial growth, as it allows the production of enzymes, structural proteins, and virulence factors. If SaTrmK gets suppressed then, without the methylation provided by SaTrmK, tRNA molecules become destabilized and may fail to correctly pair with mRNA codons during translation. This leads to errors in protein synthesis, producing dysfunctional or incomplete proteins that cannot perform their intended roles within the cell. The suppression of SaTrmK, and the resulting disruption in protein synthesis, leads to a decrease in the production of various virulence factors. Consequently, the bacterium’s ability to cause disease is significantly weakened, reducing the severity of infection. Over time, the accumulation of protein synthesis errors and the inability to produce essential proteins lead to the collapse of critical cellular processes, ultimately causing bacterial cell death. In the host, this would contribute to the clearance of the infection^47^.

*Aegle marmelos* extracts exhibit a wide range of pharmacological effects, encompassing anti-cancer, anti-inflammatory, and antioxidant properties. Recent investigations have focused on utilizing *A. marmelos* as functional food components, exploring their potential benefits and their ability to provide protection during radiation therapy^9^. Moreover, the plant demonstrates promising healing properties for ulcers and contains phenolic compounds that possess antioxidant activity. The anti-inflammatory properties of *Aegle marmelos* are another important aspect of its pharmacological profile. The plant’s extracts have been shown to reduce inflammation by inhibiting the production of pro-inflammatory cytokines and enzymes, such as cyclooxygenase (COX) and lipoxygenase (LOX). These effects are particularly valuable in the treatment of inflammatory conditions such as arthritis, colitis, and asthma. Additionally, the presence of coumarins and alkaloids in *Aegle marmelos* contributes to its ability to modulate the immune response, further enhancing its anti-inflammatory potential. The hypoglycemic effects of *Aegle marmelos* are primarily attributed to compounds like aegeline and marmesin, which modulate the activity of key enzymes involved in carbohydrate metabolism. Regular consumption of *Aegle marmelos* extracts has been reported to lower blood glucose levels and improve lipid profiles in diabetic patients^10^. Until now, there is no report on the therapeutic potential of proteins or peptides from the Bael fruit.

In this study, we will be utilizing various computational tools to design and predict the antimicrobial peptides from *Aegle marmelos* (Indian Bael) based on machine learning algorithms. Further, a molecular docking for two target proteins, dynamics simulation and MMPBSA analysis was performed to understand the potential of predicted AMPs against drug-resistant *Staphylococcus aureus*. In this study, we focus on evaluating the efficacy of the AM1 peptide against different strains of *Staphylococcus aureus*: Methicillin-Sensitive *Staphylococcus aureus* (MSSA), Methicillin-Resistant *Staphylococcus aureus* (MRSA) and Multi-Drug Resistant *Staphylococcus aureus* (MDR-SA). MSSA refers to strains of *S. aureus* that are susceptible to methicillin and other beta-lactam antibiotics, which are commonly used in clinical settings. Conversely, MRSA strains have developed resistance to methicillin, posing a major challenge in the treatment of staphylococcal infections due to limited therapeutic options. Similarly, MDR-SA refers to the strains that are resistant to broad-spectrum antibiotics and they poses major threat. The emergence of MRSA and MDR-SA has heightened the need for alternative antimicrobial agents, such as the antimicrobial peptide, which could potentially overcome antibiotic resistance mechanisms.

## Methodology

### In silico hydrolysis and prediction of AMP from the parent protein

Proteome sequences were collected from the UniProt database with the keyword “*Aegle marmelos*”. It was further subjected to an Expasy peptide cutter server [https://web.expasy.org/peptide_cutter/], and digested with five different enzymes Trypsin, Chymotrypsin-high specificity (C-term to [FYW], not before P), Chymotrypsin-low specificity (C-term to [FYWML], not before P), Pepsin (pH 1.3), and Pepsin (pH > 2) for the collection of shorter peptide sequences; which were further evaluated for the AMP property. These enzymes cut the proteome sequences at specific regions resulting in different lengths of peptide sequences and the benchmark was set for the sequence range from 6 to 25 amino acids length^11^. These specific enzymes were selected for the digestion of protein sequences are because of the specific cleavage patterns. The other important reasons for the selection of specific peptides are because of their presence in human body, which means that if the protein sequences is digested from these particular enzymes then if they are delivered it may not be undergoing any further cleavage in the human body. Thus protecting from the cleavage into further shorter peptides sequences and losing its activity and efficiency. Enzyme cleavage specificity for trypsin is that it cleaves at the carboxyl side of lysine (K) and arginine (R) residues, Chymotrypsin (High Specificity) **c**leaves at the carboxyl side of phenylalanine (F), tyrosine (Y), and tryptophan (W) residues, except when proline (P) precedes the cleavage site; Chymotrypsin (Low Specificity) cleaves at the carboxyl side of a broader range of hydrophobic residues, including phenylalanine (F), tyrosine (Y), tryptophan (W), methionine (M), and leucine (L); Pepsin (pH 1.3) cleaves preferentially at the N-terminal side of aromatic and hydrophobic amino acids, and Pepsin (pH > 2) has broader specificity at a higher pH, cleaving at additional sites compared to its activity at pH 1.3 respectively. The digestion of the protein sequences yielded over 1000 peptide fragments. To focus on peptides that are more likely to exhibit antimicrobial properties, a filtering criterion was applied. Only peptides with a length of 6 to 25 amino acids were selected for further analysis. This step reduced the number of potential AMP candidates to less than 50 peptides. The Database of Antimicrobial Activity and Structure of Peptides (DBAASP) server [https://dbaasp.org/tools?page=linear-amp-prediction] was used for the prediction of AMP from the collected shorter peptide sequences. Based on machine learning algorithm and Moon and Fleming scale, it predicts the antibacterial property of the linear peptide in general and against specific bacterial strain^12^.

## Physicochemical properties of the predicted AMPs

Physicochemical properties of the predicted peptides were calculated using two web servers such as APD3 [https://aps.unmc.edu/prediction], and Expasy PROTPARAM [https://web.expasy.org/protparam/], which predicted the various physiochemical properties such as molecular weight, theoretical pI, amino acid composition, atomic composition, extinction coefficient, estimated half-life, instability index, aliphatic index and grand average of hydropathicity (GRAVY)^13^. The Toxicity property of the predicted AMP was calculated using ToxIBTL (https://server.wei-group.net/ToxIBTL/Server.html), which works on the principle of a novel deep learning framework by using the principle of bottleneck and transfer learning algorithm for the toxicity prediction of the peptides^14^.

## ADMET property for the predicted AMPs

To evaluate the ADMET properties of the predicted peptides, we converted the amino acid residues to SMILES (Simplified Molecular Input Line Entry System) format using the PepSMI server (https://www.novoprolabs.com/tools/convert-peptide-to-smiles-string). SMILES, which stands for Simplified Molecular Input Line Entry System, is a notation system utilized for computationally constructing constrained peptides in the form of a text string. It precisely defined every atom and bond in the molecule, making it easily processable by server for the defined usage. The SMILES was used as an input for the prediction of Absorption, Distribution, Metabolism, Excretion and toxicity (ADMET) properties using ADMETlab 2.0 server (https://admetmesh.scbdd.com/)^15^. The key parameter that was inferred for the ADMET profile is Human Intestinal Absorption (HIA), Caco-2 permeability, volume distribution, mutagenicity, carcinogenicity, Central nervous system (CNS) permeability, Drug-Induced liver injury (DILI), different cytochrome enzymes inhibition and substrate, and skin sensitization. Overall, the AMPs with the best ADMET profiles were then, prepared for the molecular docking exercises.

## Evaluation of cell penetrating, hemolytic, toxicity, and Allergenicity Property

Cell penetrating property of the peptides were predicted using Support Vector Machine based algorithm-based web server CellPPD (https://webs.iiitd.edu.in/raghava/cellppd/index.html). Peptide sequences were subjected to amino acid residues scanning tool with the default SMV threshold and the results were obtained as CPP and Non-CPP on the basis of the prediction score^16,17^. Hemolytic prediction for the peptide’s sequences were carried out using SVM based web server HemoPI (https://webs.iiitd.edu.in/raghava/hemopi/design.php). PROB score is the normalized SVM score and ranges between 0 and 1, i.e. 1 very likely to be hemolytic, 0 very unlikely to be hemolytic^18^. Toxicity and allergenicity for the peptide were predicted using ToxinPred (https://webs.iiitd.edu.in/raghava/toxinpred/algo.php) and AllerTop server (https://www.ddg-pharmfac.net/AllerTOP)^19–21^. pH-dependent folded and unfolded states were predicted using SVM-based DispHScan (http://disphscan.ppmclab.com/)^22^.

## Structure prediction

The generated sequences for the predicted antimicrobial peptides will be modelled using PEPFOLD2.0 (https://bioserv.rpbs.univ-paris-diderot.fr/services/PEP-FOLD2/), for the 3D- dimensional structure prediction^23^. It works on the principle of HMM model (Hidden Markov Model) to predict the 3D- structure of the peptides and the peptide length should not be less than 9 amino acid residues^24^. Structure prediction of the antimicrobial peptides was performed using the PEP-FOLD server, which utilizes the sOPEP (optimized potential for efficient structure prediction) force field. The sOPEP force field is specifically designed for coarse-grained simulations of peptide folding, providing a reliable balance between computational efficiency and structural accuracy. It has been validated in numerous studies for predicting the native-like conformations of peptides and small proteins, making it suitable for our analysis of the AM1 peptide and other predicted antimicrobial peptides. The best ranked model from the results will be further validated using PROCHECK server of SAVES v6.0 with the help of Ramachandra plot by the positioning of amino acids residues in the different regions such as allowed, favoured and disallowed regions in the plot Laskowski^25^.

### Molecular Docking

**HADDOCK** (**H**igh **A**mbiguity **D**riven protein-protein **DOCK**ing) is an information-driven flexible docking approach for the modelling of biomolecular complexes. Two protein targets were selected to investigate in bidirectional suppression of the infectivity cycle of the drug-resistant *S. aureus*. Dihydrofolate reductase (DHFR) enzyme and m^1^ A22-tRNA methyltransferase (TrmK) protein was retrieved from the UniProt Database. Proteins were prepared for the docking by removing the water molecules and other heteroatom molecules and adding hydrogen bonds and charges were added using AutoDock tool. Energy minimization was done using SWISSPDB viewer tool. Similarly, peptide was prepared using AutoDock tools and energy minimized for docking study. Then molecular docking was performed using HADDOCK server and protein-peptide docking was performed specific to the active site residues^26,27^. To compare the binding efficiency of the peptides against the target protein, trimethoprim was as a standard drug control and has been docked against the target protein of *S. aureus*. The active site for the target protein was predicted from the Computed Atlas of Surface Topography of Proteins (CASTp 3.0 version) (http://sts.bioe.uic.edu/castp/calculation.html), and from the literature previously reported for the protein binding site^45,47^. The output results were recorded for different parameters such as HADDOCK score, Van der Waals energy, Electrostatic energy, and Desolvation energy. Interaction visualization between protein-peptide complex were studied using BIOVIA Discovery Studio and LigPlot tools.

## Protein-peptide interface interaction analysis

To study the protein-peptide interface interactions, PRODIGY web server (https://rascar.science.uu.nl/prodigy/) was employed, it predicts the binding energy for the docked protein-peptide complexes in Kcal/mol unit. The binding energy evaluated at the PRODIGY server, was calculated using following Eq. 2^28^.





Where, ICs (Inter-residue contacts) and NIS (Non-interacting surface) terms represent the importance of different types of residues interaction in defining the overall binding energy for the interacted complex.

## Molecular dynamics (MD) simulation

Molecular dynamics (MD) simulations were performed on protein-ligand complexes, specifically (1) the chosen protein target and peptides, and (2) the chosen protein target and a known drug molecule that binds to the protein target. These simulations were conducted using the Charmm force field in the GROMACS package (version 2023.1)^29^. The initial structure for each protein-ligand complex was derived from molecular docking results. The force field for each ligand was created using the Charmm-GUI server and the CHARMM36m force field. The complexes were solvated using the TIP3 solvent model, Monte-Carlo ion placement method, and Na + or Cl- counterions to neutralize the system. Each simulation underwent energy minimization via the steepest descent method, followed by a 1 ns equilibration run where each structure was gradually heated from 100 K to 310.15 K. Subsequently, each equilibrated structure was subjected to a 300 ns productive MD run in the NPT ensemble at 310.15 K and 1 atm pressure. The temperature was regulated using the velocity-rescale algorithm, while the pressure was controlled by the Berendsen barostat. The Particle-Mesh-Ewald (PME) method was used to set up the periodic boundary condition for the explicitly solvated systems, and a 1-nm cut off distance was applied for short-range interactions. The P-LINC algorithm was used to apply geometric constraints to the covalent bonds involving hydrogen atoms, allowing a 2-fs timestep size. After all simulations were completed, water molecules and translational and rotational motions were removed from the trajectory. Only the internal motions of the protein-ligand complex were analysed. The root means square deviation (RMSD) was computed from each simulation to monitor the overall conformational changes of the protein and the ligand. The per-residue root means square fluctuations (RMSF) were calculated to evaluate the local flexibility and rigidity induced by ligand binding.

### The molecular mechanics/Poisson–Boltzmann surface area (MM-PBSA)

The interaction free energies of protein-peptide complex were determined using the gmx_MMPBSA technique, which is a quantitative calculation of the binding free energy used to examine bio-molecular complexes^30^. The binding energy was calculated for the known standard and the predicted peptide bound at the active site of the target protein. The MD simulation trajectories for the 100 ns simulation were utilized to calculate the binding energy of the complexes. Other interaction analysis such as ΔVDWAALS, ΔEEL, ΔEPB, ΔENPOLAR, ΔGGAS, and ΔGSOLV respectively were also analysed.

### Bacterial strains and culture media

The bacterial strains Methicillin Resistant *Staphylococcus aureus* (MRSA) (ATCC 33591), Methicillin Sensitive Staphylococcus aureus (MSSA) (ATCC 25923), and Multi-drug resistant *Staphylococcus aureus* (ATCC BAA-44) were obtained from the American Type Culture Collection (ATCC). Mueller-Hinton broth (MHB), Muller-Hinton Agar (MHA), and Mannitol Salt Agar (MSA) media were purchased from Himedia Laboratories, India.

### Peptide synthesis

Peptide sequence **AM1** - **GKEAATKAIKEWGQPKSKITH** were synthesized by GenScript, Biotech Desk Pvt Ltd, India. The purity of the peptide was > 95% (confirmed by analytical Reverse-phase high-performance liquid chromatography (RP-HPLC).

### MIC and MBC determination

Minimum inhibitory concentrations (MIC) of AM1 were determined using micro-dilution broth assay according to clinical and laboratory standards institute (CLSI) guidelines with some modifications^31^. Briefly, overnight cultures were diluted in Muller-Hinton broth (MHB) to achieve a final density of 5 × 10^5^ CFU/ml. A volume of 100 µl of bacterial suspension was added to wells of sterile 96-well plates containing 100 µl of two-fold serial dilutions of peptide and Trimethoprim (antibiotic), ranging from 0.390 to 50 µg/ml. After incubation at 37 °C for 18 h, the lowest concentration of peptide at which no visible growth is seen was defined as the MIC, while the lowest concentration of antimicrobial causing at least 99.9% killing of the initial inoculums was considered as MBC. MBC was determined by removing samples from wells with no visible growth, serially diluting, and plating on Mueller Hinton (MH) agar plates for CFU counting.

### Time-kill kinetics assay

Time-kill kinetics assay was used to investigate the killing rate of AM1 peptide and antibiotic (Trimethoprim) against *Staphylococcus aureus* strains. For each strain, a suspension with a final density of 10^5^ CFU/ml was prepared from overnight cultures and added to each well of the 96-well microtiter plate in the presence of peptide (1X MIC, and 2X MIC). The peptide-free control was included for each strain. Following incubation at 37 °C for different time intervals, each sample was serially diluted, plated onto Mueller–Hinton (MH) agar plates and incubated at 37 °C. The viable plate count method was performed after incubation for 24 h and the counts were expressed as Log CFU/ml^32^.

### Stability

To detect the influence of salt ions, pH, and temperature, on the antibacterial activity of AM1, 1X MIC of AM1 peptide was incubated with different concentration NaCl, KCl, CaCl2, and MgCl2 solutions (0, 50, 100, 150, and 200 mmol/mL) for 30 min. Solutions with different pH values were prepared with HCl and NaOH, producing pH values of 4, 5, 6, 7, 8, 9, 10, and AM1was incubated in these solutions for 30 min. AM1 solutions were also incubated at different temperatures for 30 min (0, 25, 50, 75, and 100 °C). 100 µl of each of the peptide solutions treated as described above was collected, incubated with the different bacterial strains (MSSA, MRSA, and MDR-SA strains), and the changes in the antimicrobial activity of the AMPs were detected at OD 600 nm^33^.

### Statistical analysis

All experiments were performed in triplicate and repeated three times. Data were represented as mean ± SD from three independent experiments and analyzed by Graph-Pad Prism software version 9.5 (San Diego, CA, United States).

## Results

### In silico hydrolysis and prediction of AMP from the parent protein

Protein sequences were retrieved from the UniProt database for the *Aegle marmelos* (Indian Bael fruit) and subjected to expasy peptide cutter tool for cleavage of larger protein sequences into shorter peptide sequences using different enzymes (Table 1). Totally Five enzymes which were used in the cleaving the protein sequences of *A. marmelos*. From 29 protein sequences from the UniProt database, when digested using different enzymes, we got only 51 protein sequences which upon cleaving resulted in shorter peptides sequences that showed antimicrobial peptide property. When protein sequences were cleaved with trypsin enzyme, 9 shorter peptide sequences were obtained that showed AMP property. Similarly, when cleaved using chymotrypsin with low specificity, 9 shorter peptide sequences; using high specificity 14 shorter peptide sequences, using Pepsin pH 1.3, 13 shorter peptide sequences; and using pepsin pH > 2, 6 shorter peptide sequences were obtained after analyzing through the DSAABP server for the prediction of antimicrobial peptide property.

**Table 1:** Protein sequences of *Aegle marmelos* and different enzymes used for the cleavage with their cleaved shorter peptide sequences.

Protein sequences	Trypsin	Pepsin (pH 1.3)	Pepsin (pH > 2)	Chymotrypsin-low specificity (C-term to [FYWML], not before *P*	Chymotrypsin-high specificity (C-term to [FYW], not before *P*)
Ribulose-1,5-bisphosphate carboxylase/oxygenase large subunit, partial (chloroplast)		KAGVKDYK		KAGVKDY	
AEGMA Maturase K	NLSHYYSGSSK	KRLGSE SVKRLITRMYQRINL	SVKRLITRM	SCVKSL VRICRNL	HVSSQPGRVQLNHLY VRICRNLSHY DNKSSSLSVKRLITRMY
AEGMA Ribulose bisphosphate carboxylase large chain (Fragment)		GHPWGNAPGAVANRVAL	HIHRAMHAVIDRQKNHGMH	AGTVVGKL	
AEGMA Ribosomal protein S4 (Fragment)		KKIRRLGAL		KKIRRL	
AEGMA ATP synthase subunit beta (Fragment)	MPNIYNALVVK TTSPIHK	TGSPGKYVG		GNNRVRAVAM INNIAKAH	RRGGKIGLF ETAQRVKQTLQRY
AEGMA Ribulose bisphosphate carboxylase large chain (Fragment)		SAKNYGRAVYEC FMRWRDRF			QGPPHGIQVERDKLNKY
AEGMA Malate dehydrogenase (Fragment)		DAVKVIKPTI			GLIVSSRKDSLQHF
AEGMA Miraculin-like protein 2 (Fragment)	LCNSVGR				KEGVRRLVVV CNSVGRF KEGVRRL
AEGMA Beta-hydroxylase (Fragment)		WARWAHKALWHASL			AHKALW
AEGMA Maturase K (Fragment)	LNSLLVR FCNALGHPISK	KDPFMHYVRYQGKSIL			NSNSLITSKNSISKSNPRLLLF CNALGHPISKSTW
AEGMA Xanthine dehydrogenase (Fragment)	CTGYRPIVDAFR WYRPLK		RKVVTERPAH IVGKSW		
AEGMA Type III polyketide synthase		GKEAATKAIKEWGQPKSKITH KASRHVMSE	GKEAATKAIKE WGQPKSKITH	GKEAATKAIKEW GQPKSKITH	GQPKSKITHLIF

Initially, when different enzymes were used for the cleavage of protein sequences into shorter peptide, more than 1000 shorter sequences were obtained compiling all the protein sequences. Later, the basis of window size of amino acid residue size of 6–25 were applied, number of shorter sequences got reduced to less than 300 number. Further, when those shorter peptide sequences were given as input to the DSAABP server for the prediction of AMP property. From the server, we predicted 51 peptides sequences to have the antimicrobial property, for which prediction was performed based on the machine learning algorithm and uses the Moon and Fleming scale^34,35^.

### Physicochemical properties of the predicted AMPs

Physicochemical properties for the predicted antimicrobial peptides were predicted using different webservers such as APD3 antimicrobial database and PROTPARAM. The properties include various characteristics such as Peptide mass, charge, pI (isoelectric point), Hydrophobicity value, Hydropathy value, Boman index, Instability index, and half lifetime. To streamline the study and enhance the focus, we have prioritized the peptides with the maximum half life time and stability index. Out of 51 peptides obtained from digesting from different enzymes, we took 15 peptides for further studies on the basis of physiochemical properties such as hydrophobicity value, net charge value, isoelectric point (pI) and other properties. The physiochemical properties for the 15 peptides are given below and the rest peptides physiochemical properties are given as supplementary information in the Table S1 (a & b). Lowest and highest peptide length obtained were 9 and 22 amino acid residues with the peptide sequence GQPKSKITH, TGSPGKYVG, and NSNSLITSKNSISKSNPRLLLF and peptide mass of 995.15, 864.96, and 2433.79 respectively. For peptide sequence SVKRLITRMYQRINL, net charge – 4 is maximum, whereas the pI value 12.00 is maximum for GNNRVRAVAM among all the predicted AMP sequences. Hydrophobicity value for the peptide sequence GKEAATKAIKEWGQPKSKITH is maximum with 8.66 value and TGSPGKYVG has lowest value with 0.87. Hydrophobicity value is the sum of whole-residue free energy of transfer of the peptide from water to POPC interface and termed as Wimley-white whole residues value (Table 2a).

**Table 2:** Physicochemical properties for predicted antimicrobial peptides.

Peptide codes	Peptide Sequence	Peptide Length	Peptide mass (Daltons)	Charge	pI	Hydrophobicity (Wimley-White whole-residue)	Hydropathy value	Boman Index (kcal/mol
I	SVKRLITRMYQRINL	15	1891.31	+ 4	11.72	1.85	-0.3	2.83
II	GNNRVRAVAM	10	1087.27	+ 2	12.00	2.72	-0.25	2.81
III	SAKNYGRAVYEC	12	1360.51	+ 1	7.90	2.67	-0.7416	2.31
IV	GKEAATKAIKEW	12	1331.53	+ 1	8.50	5.51	-0.9	1.6
V	GQPKSKITHLIF	12	1368.64	+ 2.25	10.00	1.15	-0.2	0.71
VI	GQPKSKITH	9	995.15	+ 2.25	10.00	3.15	-1.5	2.37
VII	GKEAATKAIKE	11	1145.32	+ 1	8.50	1.3	-1.44	1.9
VIII	WGQPKSKITH	10	1181.36	+ 2.25	10.00	1.3	-1.44	1.9
IX	GHPWGNAPGAVANRVAL	17	1686.89	+ 1.25	9.76	1.16	-0.03529	0.43
X	QGPPHGIQVERDKLNKY	17	1979.23	+ 1.25	8.50	6.97	-1.5176	2.84
XI	NSNSLITSKNSISKSNPRLLLF	22	2433.79	+ 3	11.17	1.85	-0.2909	1.95
XII	CTGYRPIVDAFR	12	1397.61	+ 1	8.22	1.07	-0.05833	2.1
XIII	GKEAATKAIKEWGQPKSKITH	21	2308.67	+ 3.25	9.83	8.66	-1.1571	1.93
XIV	TGSPGKYVG	9	864.95	+ 1	8.26	0.87	-0.5888	0.53
XV	DAVKVIKPTI	10	1083.34	+ 1	8.59	3.49	0.56	0.26

**Table 3:** Instability index and the half-life time for the predicted antimicrobial peptides.

Peptide Sequence	Instability Index	Half Life Time		
		Mammalian reticulocytes (in-vitro)	Yeast (in-vivo)	Escherichia coli (in-vivo)
SVKRLITRMYQRINL	44.93 - Stable	1.9 h	> 20 h	> 10 h
GNNRVRAVAM	0.51 - Stable	30 h	> 20 h	> 10 h
SAKNYGRAVYEC	26.14 - Stable	1.9 h	> 20 h	> 10 h
GKEAATKAIKEW	-17.51 - Stable	30 h	> 20 h	> 10 h
GQPKSKITHLIF	18.14 - Stable	30 h	> 20 h	> 10 h
GQPKSKITH	20.86 - Stable	30 h	> 20 h	> 10 h
GKEAATKAIKE	-6.35 - Stable	30 h	> 20 h	> 10 h
WGQPKSKITH	9.40 - Stable	2.8 h	3 min	2 min
GHPWGNAPGAVANRVAL	1.26 - Stable	30 h	> 20 h	> 10 h
QGPPHGIQVERDKLNKY	9.15 - Stable	0.8 h	10 min	10 h
NSNSLITSKNSISKSNPRLLLF	4.81 - Stable	10 h	> 20 h	> 10 h
CTGYRPIVDAFR	4.54 - Stable	6 h	> 20 h	> 10 h
GKEAATKAIKEWGQPKSKITH	-5.53 - Stable	30 h	> 20 h	> 10 h
TGSPGKYVG	29.41 - Stable	7.2 h	> 20 h	> 10 h
DAVKVIKPTI	-18.40 - Stable	1.1 h	3 min	> 10 h

Boman index provides the possible protein interaction index based on amino acid residues present in the protein^35^. It calculates based on solubility properties of all the amino acid residues in each peptide sequences by dividing the total number of residues for normalizing. Therefore, it provides the probability of peptide to bind to a protein or membrane as a receptor. Peptide sequence with SVKRLITRMYQRINL has highest boman index with 2.83 (Kcal/mol) and sequence DAVKVIKPTI has lowest boman index value with 0.26 (Kcal/mol).

**Table 4:** Predicted ADMET scores for the antimicrobial peptides.

Category	Property	I	II	III	IV	V	VI	VII	VIII
Absorption	HIA/Human Intestinal absorption (%)	0.989	0.306	0.926	1	0.996	0.972	1	0.978
	Papp/Caco-2 permeability (cm/s)	-6.165	-7.143	-7.689	-7.627	-6.85	-7.096	-7.885	-6.45
Distribution	VD/Volume distribution (L/Kg)	0.351	0.506	0.548	0.542	0.501	0.521	0.506	0.48
	BBB/Blood brain barrier penetration (%)	0.012	0.08	0.032	0.036	0.026	0.068	0.05	0.056
	PPB/Plasma protein binding (%)	26.60	11.72	21.85	20.92	24.74	19.95	20.49	20.25
Metabolism	CYP1A2 – Inhibitor	0	0	0	0	0	0	0	0
	CYP1A2 – substrate	0	0	0	0	0.002	0.007	0	0.004
	CYP3A4 – Inhibitor	0	0	0.001	0	0.035	0.006	0	0.026
	CYP3A4 - substrate	0	0	0	0	0.001	0	0	0
Excretion	CL/Clearance (ml/min/Kg)	0.335	0.883	0.891	1.174	1.123	1.063	1.182	1.218
Toxicity	hERG blockers	0.003	0.007	0.012	0.001	0.004	0.005	0	0.011
	DILI/Drug induced liver Injury	0	0.009	0.004	0.001	0.005	0.004	0.001	0.003
	AMES (Ames mutagenicity)	0.004	0.008	0.02	0.004	0.004	0.009	0.005	0.007
	Carcinogenicity	0.101	0.078	0.06	0.066	0.026	0.022	0.092	0.013
	Skin sensitization	0.036	0.082	0.13	0.115	0.076	0.163	0.134	0.128
	Log P value	-0.782	-3.454	-3.631	-3.782	-0.581	-4.229	-5.034	-3.519
	H acceptor	48	31	36	34	33	27	31	30
	H donor	38	24	26	24	22	19	22	21

Note – I – VIII represents the peptide codes mentioned in the Table 2.

Further, instability index and the half lifetime of the predicted AMP were predicted using Expasy PROTPARAM server. Peptide those stable was taken for the further analysis and the stability index for the prediction was based on the range of smaller than is given as stable, whereas more than 40 is given as unstable peptide. From the half-life time, it has been observed that 7 predicted peptide sequences have equal to or greater than 30 h of half-life time in mammalian reticulocytes, in vivo, similarly for yeast, in vivo 12 peptide sequences showed the maximum half-life time of equal to or greater than 20 hours’ duration, and for Escherichia coli, in vitro, 14 peptide sequences has half-life time equal to or greater than 10 h. Peptide sequence QGPPHGIQVERDKLNKY has lowest half-life time in mammalian reticulocytes with 0.8 h (Table 2).

### ADMET property for the predicted AMPs

Evaluating the pharmacokinetic properties of the predicted peptides is important for investigating its efficacy and indemnity. In the current evaluation of ADMET property analysis, we have considered several parameters such as Human Intestinal Absorption (HIA), mutagenicity, carcinogenicity, Central Nervous System (CNS) permeability, drug induced liver injury, Cytochrome P450 enzymes inhibition, etc. Human Intestinal Absorption is one the important parameter that needs to be considered, because while administrating drug orally, HIA influences the bioavailability^36^. Based on their physicochemical properties, we identified 15 peptides that exhibited both an extended half-life in mammalian reticulocytes and a stability index indicating high stability, alongside enhanced hydrophobicity. ADMET properties were subsequently predicted for these selected peptides, which were then further characterized and utilized in molecular docking studies.

All the 15 predicted peptides have the HIA value more than 0.5, and it can be easily absorbed in the human intestine. For Blood brain barrier permeability index, BBB value more than 0.3 can result in causing the CNS toxicity, therefore BBB value less than 0.3 can be considered safe. In that consideration, all the 15 peptides have BBB index less than 0.3 value under optimal distribution volume for the peptides were in the range of 0.069–0.55 L/kg, which has to be in the range of 0.04–20 L/Kg value. For the cytochrome P450 enzymes, none of the peptides were able to inhibit the activity, thereby falling below the range of 0.5 value; which is considered as range for the no inhibition activity.

**Table 5:** Predicted ADMET scores for the antimicrobial peptides.

Category	Property	IX	X	XI	XII	XIII	XIV	XV
Absorption	HIA/Human Intestinal absorption (%)	0.876	0.997	0.971	0.99	1	0.922	0.999
	Papp/Caco-2 permeability (cm/s)	-6.24	-7.342	-7.175	-8.147	-7.566	-7.482	-7.429
Distribution	VD/Volume distribution (L/Kg)	0.43	0.55	0.069	0.471	0.169	0.511	0.513
	BBB/Blood brain barrier penetration (%)	0.04	0.015	0.007	0.029	0.014	0.055	0.029
	PPB/Plasma protein binding (%)	34.41	27.01	22.20	17.74	28.91	19.61	17.25
Metabolism	CYP1A2 – Inhibitor	0	0	0	0	0	0	0
	CYP1A2 – substrate	0	0	0	0	0	0.001	0.001
	CYP3A4 – Inhibitor	0.002	0	0	0.002	0	0.003	0.007
	CYP3A4 - substrate	0	0	0	0	0	0.001	0.003
Excretion	CL/Clearance (ml/min/Kg)	0.404	0.135	1.159	0.91	0.668	1.402	1.761
Toxicity	hERG blockers	0.002	0.001	0	0.013	0	0.016	0.001
	DILI/Drug induced liver Injury	0.044	0	0	0.02	0	0.005	0.017
	AMES (Ames mutagenicity)	0.001	0.007	0.019	0.003	0.002	0.008	0.002
	Carcinogenicity	0.013	0.1	0.153	0.11	0.014	0.035	0.139
	Skin sensitization	0.134	0.132	0.05	0.059	0.067	0.294	0.034
	Log P value	-2.556	-6.036	-3.932	-1.315	-6.159	-3.464	-0.289
	H acceptor	45	53	65	35	60	23	26
	H donor	27	35	46	24	41	15	17

Note – IX – XV represents the peptide codes mentioned in the Table 2.

Also, the clearance level in the excretion section for all the peptides are below 5 ml/min/Kg, which is considered as low clearance level therefore will not accumulate in the intestine. For toxicity property, hERG value is less than 0.5 for all the peptides, similarly for other parameters such as carcinogenicity, mutagenicity, and skin sensitization, all the peptides fall below the value which is considered as harmful for these parameters. When these peptides were subjected for PAINS (Pan Assay Interference Compounds) and BMS (Bristol-Meyers Squibb’s) reactions for screening process, there were no reactions alerts were detected resulting in inferring that these peptides are safer to use as therapeutic agents (Table 4,5).

### Evaluation of cell penetrating, hemolytic, toxicity, and Allergenicity Property

Peptides were analyzed for their various characteristics such as cell penetrating property, which can help them to penetrate through the cells and kill the bacteria. Other important property to be investigated for the peptides are hemolytic, toxicity and allergenicity property to avoid hemolysis, neglecting out toxicity nature of the peptides and not having any allergenicity towards the immune system.

**Table 6:** Different biological properties of the predicted antimicrobial peptides and their pH dependent folding states.

Peptide Sequences	Cell Penetrating	Hemolytic	Toxicity	Allergenicity	Folding states
SVKRLITRMYQRINL	Non-CPP	0.49	Non-Toxin	Non-Allergen	Conditional folding
GNNRVRAVAM	Non-CPP	0.49	Non-Toxin	Non-Allergen	Non – confident transition
SAKNYGRAVYEC	Non-CPP	0.48	Non-Toxin	Non-Allergen	Folded
GKEAATKAIKEW	Non-CPP	0.49	Non-Toxin	Non-Allergen	Conditional folding
GQPKSKITHLIF	Non-CPP	0.47	Non-Toxin	Non-Allergen	Folded
GQPKSKITH	Non-CPP	0.49	Non-Toxin	Non-Allergen	Conditional folding
GKEAATKAIKE	CPP	0.49	Non-Toxin	Non-Allergen	Conditional folding
WGQPKSKITH	Non-CPP	0.48	Non-Toxin	Non-Allergen	Conditional folding
GHPWGNAPGAVANRVAL	Non-CPP	0.48	Non-Toxin	Non-Allergen	Folded
QGPPHGIQVERDKLNKY	Non-CPP	0.43	Non-Toxin	Non-Allergen	Conditional folding
NSNSLITSKNSISKSNPRLLLF	Non-CPP	0.49	Non-Toxin	Non-Allergen	Folded
CTGYRPIVDAFR	Non-CPP	0.48	Non-Toxin	Non-Allergen	Folded
GKEAATKAIKEWGQPKSKITH	CPP	0.47	Non-Toxin	Non-Allergen	Conditional folding
TGSPGKYVG	Non-CPP	0.49	Non-Toxin	Non-Allergen	Folded
DAVKVIKPTI	Non-CPP	0.48	Non-Toxin	Non-Allergen	Folded

From the results (Table 6), it has been observed that the peptides sequences - GKEAATKAIKE and GKEAATKAIKEWGQPKSKITH have cell penetrating property whereas other peptides do not possess the CPP property. For hemolytic property, all the peptides have the value less than 0.5, which indicates that they are non-hemolytic peptides and will not hemolyse the blood cells. For toxicity and allergenicity, it has been found that all the peptides are non-toxic and non-allergen, thereby they will not have any toxic effects and also can be used for drug delivery application without any allergenicity effect. Similarly, the pH dependent folding and unfolding property in the range of pH 3–10 were predicted and it has been found that 7 peptides sequences are in folding states, whereas 7 peptides sequences are in conditional folding states, and one peptide sequences is in non-confident transition states.

#### Structure prediction

**Fig. 1:**
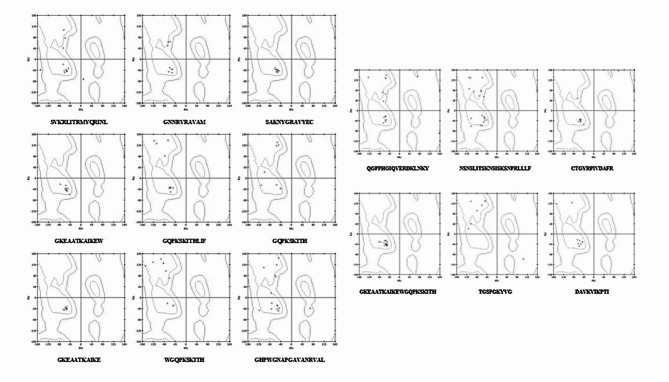
Ramachandran plot for the different predicted antimicrobial peptides representing the secondary structure property.

The 3D- structure of the antimicrobial peptides were predicted using PEPFOLD server. PEP-FOLD is a novel method designed to predict the structure of peptides by analyzing amino acid sequences. It employs a structural alphabet called SA letters, characterizing the conformations of four consecutive residues. This approach combines the predicted SA letter sequences with a greedy algorithm and coarse-grained force field to determine the peptide’s structure. It will generate maximum of 5 models for each input sequences, from which we took the best model. The models are generated based on lowest sOPEP force field (a coarse-grain energy) with the maximum melting temperature predicted, and therefore it is taken as a best model for further analysis. Further, the models generated using PEPFOLD were subjected to PROCHECK server for the structure validation using Ramachandran plot. From all the 15 peptide structures, only four peptides have all their complete residues in their most favoured regions (100%) and they are SAKNYGRAVYEC, GKEAATKAIKEW, GKEAATKAIKE, and GKEAATKAIKEWGQPKSKITH respectively. Whereas the other peptides have their residues in two regions, that is most favoured and allowed regions, but no peptides sequences have their residues in the disallowed regions (Fig. 1). Accurate structure prediction is fundamental thing for the success of computational drug design and molecular docking studies. The quality of the predicted 3D structure of a peptide directly influences the reliability of subsequent analyses, including docking, MD simulations, and free energy calculations. Accurate structure prediction ensures that the docking poses and binding interactions observed are reflective of real-time scenarios, increasing the likelihood that the identified peptides will perform effectively in experimental settings.

### Molecular docking

Molecular docking for the protein target and the predicted antimicrobial peptides were performed using HADDOCK server. Site specific docking was performed for the predicted peptides against the target protein of *Staphylococcus aureus*. Peptide sequence GKEAATKAIKEWGQPKSKITH has shown the maximum binding affinity with the HADDOCK score of -100.8 ± 7.6 and − 109.5 ± 4.7 for DHFR and TrmK target respectively. The active site or binding pocket amino acid residues for DHFR includes ILE3, ILE5, VAL6, ALA7, ILE14, GLY15, ASN18, LEU20, ILE31, LYS45, THR46, SER49, ILE50, and PHE92 respectively. Similarly, the active site residues for SaTrmK enzyme are ARG7, ASP22, GLY24, GLU47, VAL48, ASP75, GLY76, CYS92, MET94, LEU98 and ILE102 respectively. From the results it has been observed that the peptide AM1 has been able to bind to the defined active site pocket with maximum binding energy in comparison to the other peptides.

**Table 7:** Docking scores for the predicted antimicrobial peptides against *S. Aureus* DHFR protein.

	I	II	III	IV	V	VI	VII	VIII
HADDOCK score	-90.4 ± 2.8	-70.2 ± 5.0	-87.6 ± 3.4	-72.5 ± 3.5	-78.2 ± 3.4	-90.4 ± 2.0	-72.1 ± 2.1	-95.4 ± 4.1
Van der Waals energy	-53.4 ± 2.6	-48.3 ± 5.3	-44.2 ± 3.3	-43.0 ± 2.9	-39.2 ± 4.7	-53.9 ± 2.1	-50.9 ± 3.8	-55.8 ± 0.7
**Electrostatic energy**	**-131.6 ± 15.7**	**-105.0 ± 18.0**	**-140.0 ± 29.2**	**− 117.5 ± 7.2**	**-147.4 ± 27.9**	**-120.5 ± 10.8**	**-118.1 ± 14.8**	**-156.8 ± 16.7**
**Desolvation energy**	**-15.7 ± 3.2**	**-4.6 ± 1.8**	**-18.9 ± 2.1**	**-9.8 ± 1.3**	**-15.9 ± 2.3**	**-14.5 ± 1.8**	**-4.9 ± 3.0**	**-14.4 ± 1.4**

Note – I – VIII represents the peptide codes mentioned in the Table 2.

**Table 8:** Docking scores for the predicted antimicrobial peptides against *S. Aureus* DHFR protein.

	IX	X	XI	XII	XIII	XIV	XV
HADDOCK score	-93.8 ± 2.4	-67.1 ± 5.6	-67.9 ± 2.4	-92.7 ± 1.5	-100.8 ± 7.6	-75.5 ± 2.8	-67.8 ± 11.1
Van der Waals energy	-54.9 ± 3.0	-50.5 ± 2.9	-50.7 ± 1.2	-52.3 ± 0.6	-59.6 ± 2.5	-33.6 ± 4.5	-36.4 ± 5.9
**Electrostatic energy**	**-89.3 ± 18.2**	**-74.8 ± 21.4**	**-90.8 ± 10.7**	**-66.7 ± 10.2**	**-84.8 ± 25.7**	**-156.4 ± 10.3**	**-118.1 ± 31.9**
**Desolvation energy**	**-25.3 ± 4.3**	**-17.4 ± 3.0**	**-13.3 ± 1.0**	**-32.3 ± 1.8**	**-27.0 ± 1.8**	**-13.4 ± 1.3**	**-10.2 ± 0.7**

Note – IX – XV represents the peptide codes mentioned in the Table 2a.

**Fig. 2:**
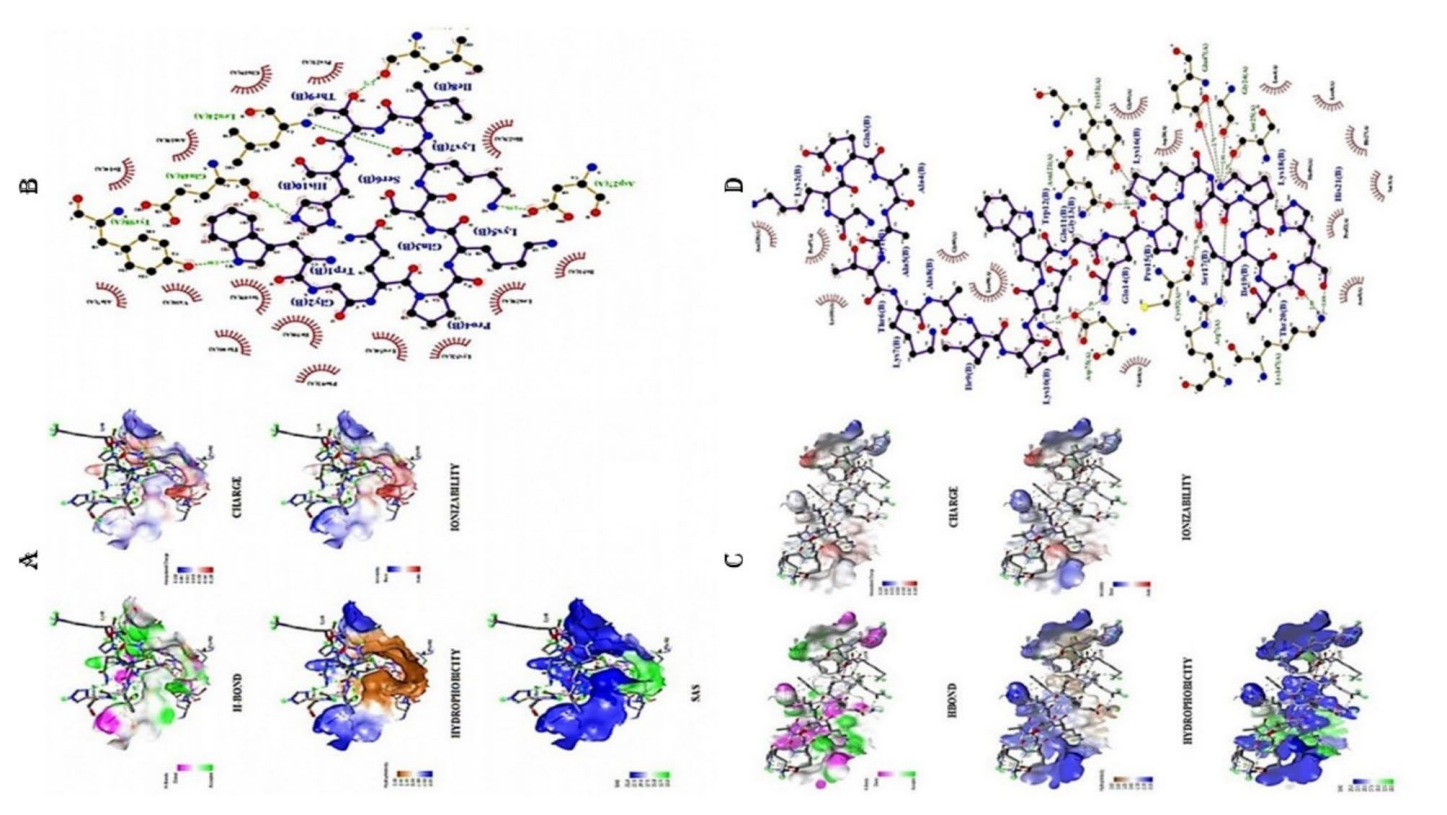

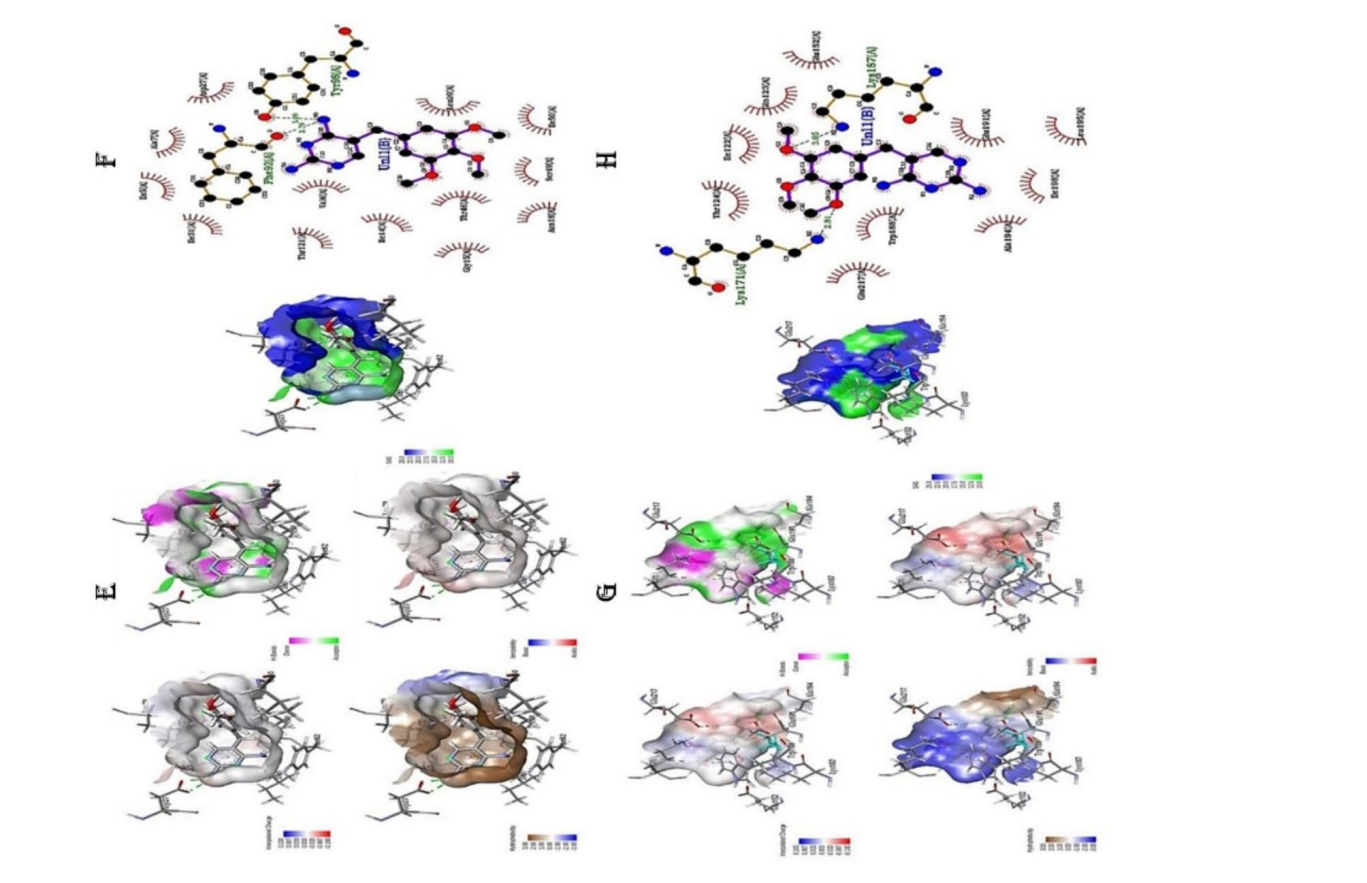
(**A & C**) Different interactions of peptide **[GKEAATKAIKEWGQPKSKITH]** with the DHFR and SaTrmK enzyme such as Hbond, charges, hydrophobicity, ionizability, SAS in the pocket were represented using BIOVIA Discovery studio software, (**B & D**) representation of the protein-peptide complex with peptide that have the maximum binding affinity towards the target protein using LigPlus software. (**E & G**) Different interactions Trimethoprim (Standard Drug) with the DHFR and SaTrmK enzyme such as Hbond, charges, hydrophobicity, ionizability, SAS in the pocket were represented using BIOVIA Discovery studio software, (**F & H**) representation of the protein-peptide complex with peptide that have the maximum binding affinity towards the target protein using LigPlus software.

Interacting atoms of the DHFR protein with the peptide, two residues ASP75 and GLU47 were able to form salt bridges, five residues ARG7, LYS147, SER25, GLY24, CYS92, ASN121, and TYR151 has formed conventional hydrogen bonds with the peptides during the interaction along with the ASP75 also able to form hydrogen bonds in parallel forming the salt bridges (Table 11). Two residues SER3, and ASN5 has formed carbon hydrogen bond with the peptides, whereas other residues such as LEU4, HIS27, LYS101, LEU98, and PRO97 formed alkyl and Pi-alkyl interaction with the peptides. Other major docking scores obtained from the HADDOCK server such as Van der Waals energy, Electrostatic energy, and desolvation energy are − 59.6 ± 2.5, -84.8 ± 25.7, and − 27.0 ± 1.8 respectively (Tables 7,5). Interaction between the protein-peptide complexes were analysed using BIOVIA Discovery studio and LigPlus for various interactions such as HBond, charges, hydrophobicity, ionizability, SAS, and 2-D interactions (Fig. 2A & B).

**Table 9:** Docking scores for the predicted antimicrobial peptides against *S. Aureus* TrmK protein.

	I	II	III	IV	V	VI	VII	VIII
HADDOCK score	-105.5 ± 3.0	-58.1 ± 1.3	-83.4 ± 1.1	-90.3 ± 5.8	-93.5 ± 3.6	-71.9 ± 1.0	-68.6 ± 8.6	-82.2 ± 2.6
Van der Waals energy	-49.1 ± 6.4	-36.4 ± 2.5	-31.7 ± 3.6	-41.4 ± 2.5	-41.4 ± 6.0	-26.5 ± 2.8	-28.2 ± 3.5	-36.1 ± 1.3
**Electrostatic energy**	**-275.1 ± 56.3**	**-123.3 ± 34.2**	**-219.8 ± 21.1**	**-183.8 ± 21.7**	**-228.2 ± 39.3**	**-234.1 ± 11.2**	**-224.4 ± 55.4**	**-172.2 ± 26.5**
**Desolvation energy**	**-3.5 ± 4.2**	**1.5 ± 3.4**	**-10.2 ± 2.5**	**-14.5 ± 1.4**	**-10.6 ± 1.8**	**1.0 ± 0.6**	**3.6 ± 3.1**	**-13.7 ± 4.0**

Note – I – VIII represents the peptide codes mentioned in the Table 2.

**Table 10:** Docking scores for the predicted antimicrobial peptides against *S. Aureus* TrmK protein.

	IX	X	XI	XII	XIII	XIV	XV
HADDOCK score	-84.9 ± 1.2	-85.7 ± 5.0	-97.8 ± 9.4	-77.3 ± 5.8	-109.5 ± 4.7	-69.3 ± 2.3	-62.9 ± 2.8
Van der Waals energy	-49.9 ± 3.6	-35.6 ± 2.7	-62.8 ± 6.9	-43.1 ± 4.2	-49.5 ± 6.9	-35.6 ± 3.5	-40.3 ± 1.2
**Electrostatic energy**	**-98.7 ± 19.7**	**-284.4 ± 40.6**	**-173.6 ± 22.7**	**-44.1 ± 18.2**	**-295.4 ± 27.9**	**-141.4 ± 14.7**	**-134.3 ± 10.0**
**Desolvation energy**	**-20.3 ± 1.6**	**0.5 ± 1.4**	**-8.7 ± 1.2**	**-28.5 ± 4.7**	**-6.9 ± 4.4**	**-6.3 ± 3.1**	**2.5 ± 1.1**

Note – IX – XV represents the peptide codes mentioned in the Table 2.

**Table 11:** Interacting atoms between the protein and AMP during molecular docking for DHFR enzyme.

Peptide Sequence	Hydrogen bonds interactions atoms	Other interactions atoms
SVKRLITRMYQRINL	Gln19, Tyr16, Gln17, Lys52, Ser49, Lys32	His23, Trp22, Leu20, Phe92, Ile50, Leu28, Leu54, Pro55, Arg57
GNNRVRAVAM	Trp22, Gln19	His23, Leu20, Ser49, Ile50, Phe92, Ile31, Leu54, Leu28
SAKNYGRAVYEC	Ile50, Lys52, Trp22	His23, Gln19, Glu48, Ser49, Leu28, Thr48, Phe92, Leu28
GKEAATKAIKEW	His23, Ile50, Ser49, Lys52	Leu28, Asp27, Leu24, Trp22, Ile31, Leu20
GQPKSKITHLIF	Gln19, Ser49, Lys32, Ile50	Leu20, Leu28, His23, Ile31, Leu54, Phe92, Lys52
GQPKSKITH	Ile50, Leu20, Gln19, Thr121, Ala7	His23, Trp22, Ser49, Asp27, Ile31, Leu28, Phe92, Gly94, Ile14, Gly15, Leu24, Val6, Tyr98
GKEAATKAIKE	Asn18, Ile50, Leu20, Lys32	Gln19, Ser49, Trp22, His23, Leu28, Ile31, Lys52, Pro55, Leu54
WGQPKSKITH	Lys52, Ile50	Phe92, Thr46, Ile50, Ser49, Val6, Ala7, Ile14, Asn18, Gln19, Pro21, His23, Ile31, Leu28, Lys52, Leu54
GHPWGNAPGAVANRVAL	Lys32, Lys52	His23, Trp22, Leu24, Ser49, Leu28, Leu20, Leu54, Pro53, Ile50, Ile31, Pro55
QGPPHGIQVERDKLNKY	Leu20, Ser49	Lys52, Phe92, Leu28, His23, Asn18, Ile31, Thr46, Ile50, Gln19
NSNSLITSKNSISKSNPRLLLF	Trp22, Leu20, Gln18, His23, Glu143, Thr146	Phe117, Lys144, Ile147, Tyr16, Glu17, Gln118, Pro21
CTGYRPIVDAFR	Lys32, Trp22	Ile31, Ile50, Gln19, Leu20, His23, Pro25, Lys29, Pro21, Leu28
GKEAATKAIKEWGQPKSKITH	Asp27, Leu20, Leu24, Glu48, Tyr98	Ser49, Trp22, Leu28, His23, Leu20, Gln19, Pro21, Tyr16, Gln118
TGSPGKYVG	Lys52, Asp27	Pro55, Pro53, ile50, Thr46, Ser49, Phe92, Val6, His23, Leu20, Leu28, Ile31, Ala7
DAVKVIKPTI	Gln19, Lys52, His23	Trp22, Leu20, Ser49, Leu28, Ile50
Trimethoprim	Phe92, Tyr98	Ile5, Val6, Ala7, Ile14, Gly15, Asn18, Leu20, Asp27, Ile31, Thr46, Ser49, Ile50, Thr121

**Table 12:** Interacting atoms between the protein and AMP during molecular docking for SaTrmK enzyme.

Peptide Sequences	Hydrogen bond interaction atoms	Other interaction atoms
SVKRLITRMYQRINL	Cys92, His149, Gly24, Ser25, Glu47, Asp75	Ile49, Val48, Leu98, Asn121, Gly93, Pro52, Met94, Gly95, Leu98
GNNRVRAVAM	Lys147, Asp26, Asp75	His149, Arg7 Tyr151, Leu98, Met94, Gly93, Val48, His27, Ile49, Pro52, Gly24, Cys92, Tyr29, Asn121
SAKNYGRAVYEC	Lys147, Asp26, Gly24, Ser26, Asp75	Gly93 Ile102, Met94, Gly76, Leu98, Ile122, Tyr151, Asn121, His149, Arg7
GKEAATKAIKEW	Lys147, His27, Asp26	Asn5, Glu146, Arg7, His149, Gly24, Tyr151, Ile49, Val48, Pro52, Asn121
GQPKSKITHLIF	Asp75, Asn121, His27, Tyr151, Lys147, Cys92, Gly24, Arg7, Lys147	Ile102, Met94, leu98, His149, Gly93, Val48, Pro52, Ser25, Tyr29, Asp26
GQPKSKITH	Gly95, Gly24, Asp26, Gly46, Gln47, Asp75	Ile122, Met94, Leu98, Ile102, Gly93, Cys92, Val48, Ile49, Pro52, His27, Ser25
GKEAATKAIKE	Asp75, Gly24, Asp26, Ser25	Gly92, Pro52, Ile49, Gly95, Val48, Leu98, Met94, Asp105
WGQPKSKITH	Ser25, Gln47, His27, Tyr161, Cys92, Asn121, Lys147, Asp26	Pro52, Asn5, Arg7, Gly24, Gly95, Leu98, Gly93, Met94, Val48
GHPWGNAPGAVANRVAL	His27	Leu98, Ile49, Gln47, Cys92, Pro52, Gly93, Ser25, Asp26, Asn121, Val48, Tyr151, Arg7
QGPPHGIQVERDKLNKY	Asp26, Cys92, Gln193	Pro52, Val48, Ile49, Gly24, Gly93, Met94, Ala194, Arg190, Ile122, Asn121, Tyr151, His149
NSNSLITSKNSISKSNPRLLLF	Gln123, Lys147, Cys92, Gly24, Asp26, Arg7	Val48, Ile49, Ser25, Tyr151, His149, Ala194, Leu98, Gly95, Ile122, Ala121
CTGYRPIVDAFR	Asp26	Lys147, His149, Tyr151, Asn121, Ile150, Gly24, Cys82, Met94, Gly93, Gly95, Leu98
GKEAATKAIKEWGQPKSKITH	Ser25, Asp26, Gly24, Asn121, Arg210	Lys147, Arg7, His27, Asn5, Tyr151, Met94, Gly93, His149, Ile122, Lys201, Ala194, Ile198
TGSPGKYVG	Asn121, Gly93, Cys92, Gly76, Asp75	Gly24, Ser25, Pro52, His27, Ile102, Leu98, Met94, Val48. Ile49
DAVKVIKPTI	Asp26, Lys147, Gly24	Arg7, His149, Tyr151, Gly93, Cys92, Pro52, His27, Asn121
Trimethoprim	Lys171, Lys187	Ile122, Gln123, Thr124, Glu152, Trp188, Glu191, Ala194, Leu195, Ile198, Glu217

Interacting atoms of the protein with the peptide sequence (GKEAATKAIKEWGQPKSKITH), only one residue ASP27 form salt bridge, five residues GLU48, LEU20, LEU24, SER49, and TYR98 form conventional hydrogen bond, and ILE50, HIS23, and ASN18 form carbon hydrogen bond. Whereas other residues ILE14, ALA7, LYS52, LEU28, and PHE92, forms alkyl, Pi-alkyl, and Pi-Pi-shaped interactions respectively (Table 12). Other major docking scores obtained from the HADDOCK server for peptide-protein interactions such as Van der Waals energy, Electrostatic energy, and desolvation energy are − 49.5 ± 6.9, -295.4 ± 27.9, and − 6.9 ± 4.4 respectively (Tables 9, 10). Interaction between the protein-peptide complexes were analysed using BIOVIA Discovery studio and LigPlus for various interactions such as hbond, charges, hydrophobicity, ionizability, SAS, and 2-D interactions (Fig. 2C & D). This is the first time, *S. aureus* TrmK enzyme was used as a target for study because of its unique properties difference from the other target enzymes.

Molecular docking was performed for the protein-standard drug (Trimethoprim) and the binding energy were found to be -7.4 and − 6.8 Kcal/mol for DHFR and SaTrmK enzyme respectively. Further interacting atoms of the Trimethoprim – DHFR complex that forms the hydrogen bonds are Phe 92 and Tyr 98 and other non-hydrogen bond forming residues are 13 residues that forms salt bridges and non-covalent interactions. Similarly, for Trimethoprim-SaTrmK complex interacting atoms that forms the hydrogen bonds are Lys 171 and Lys 187, and other non-hydrogen bonds forming atoms are 10 amino acids residues that forms salt bridges and other interactions (Fig. 2E, F, G & H).

#### Protein-peptide interface interaction analysis

PRODIGY server was used for the calculation of binding energy between the docked complex of the protein-peptide. For the docking against DHFR, the maximum binding energy obtained for the peptides (XIII) is -10.2 (kcal/mol-1), which further confirms the results obtained from the HADDOCK docking where the cluster energy being obtained was maximum for the similar peptide sequence. Other interactions energy which were obtained from the analysis includes the intermolecular contacts for the charged-charged residues, charged-polar, charged-apolar, polar-polar, polar-apolar, and apolar-apolar are 5, 5, 13, 1, 15, and 16 respectively; and non-interacting surface energies for charged and apolar are 26.72, 37.45 respectively (Table 13).

**Table 13:** Protein-peptide interface interaction analysis for DHFR and SaTrmK protein.

Peptide sequences code	ΔG (kcal mol-1)		Kd (M) at ℃	
	DHFR	SaTrmK	DHFR	SaTrmK
**I**	-7.0	-9.6	7.7e-06	3e-08
**II**	-7.6	-9.0	2.7e-06	2.5e-07
**III**	-7.9	-9.2	1.5e-06	1.7e-0.7
**IV**	-7.1	-8.8	5.9e-06	3.6e-0.7
**V**	-6.9	-9.2	8.7e-06	1.7e-08
**VI**	-9.7	-9.3	3.5e06	1.5e-07
**VII**	-8.5	-7.9	6e-07	1.5e-06
**VIII**	-7.4	-8.3	31e-08	8.9e-07
**IX**	-7.7	-7.6	2.4e-06	2.8e-06
**X**	-8.9	-9.8	3.1e-07	6.5e-08
**XI**	-7.8	-9.4	1.8e-06	1.2e-07
**XII**	-7.3	-7.0	4.2e-06	8e-06
**XIII**	-10.2	-10.2	3.7e-06	1.4e-07
**XIV**	-7.6	-7.3	2.5e-06	4.4e-06
**XV**	-7.3	-7.8	4.1e-06	2e-06

Note – Peptide codes are mentioned in the Table 2.

Similarly, for the docking against *S. aureus* TrmK (*Sa*TrmK) target, the maximum binding energy obtained for the peptides is -10.2 (kcal/mol-1), which further correlates with the results obtained from the HADDOCK docking where the cluster energy being obtained was maximum for the similar peptide sequence code (XIII). Other interactions energy which were obtained from the analysis includes the intermolecular contacts for the charged-charged residues, charged-polar, charged-apolar, polar-polar, polar-apolar, and apolar-apolar are 8, 6, 24, 0, 6, and 12 respectively; and non-interacting surface energies for charged and apolar are 29.12, and 37.91 respectively (Table 13).

#### Molecular dynamics simulation

From the docking studies peptide-protein complex with the best interaction and energy docked conformations results were utilized for molecular dynamic simulation study. A 300 ns molecular simulation was performed for studying the stability of the protein before and after binding with the peptide using Gromacs-2023.1 version.

#### Analysis of the peptide against DHFR protein complex simulation

**Fig. 3:**
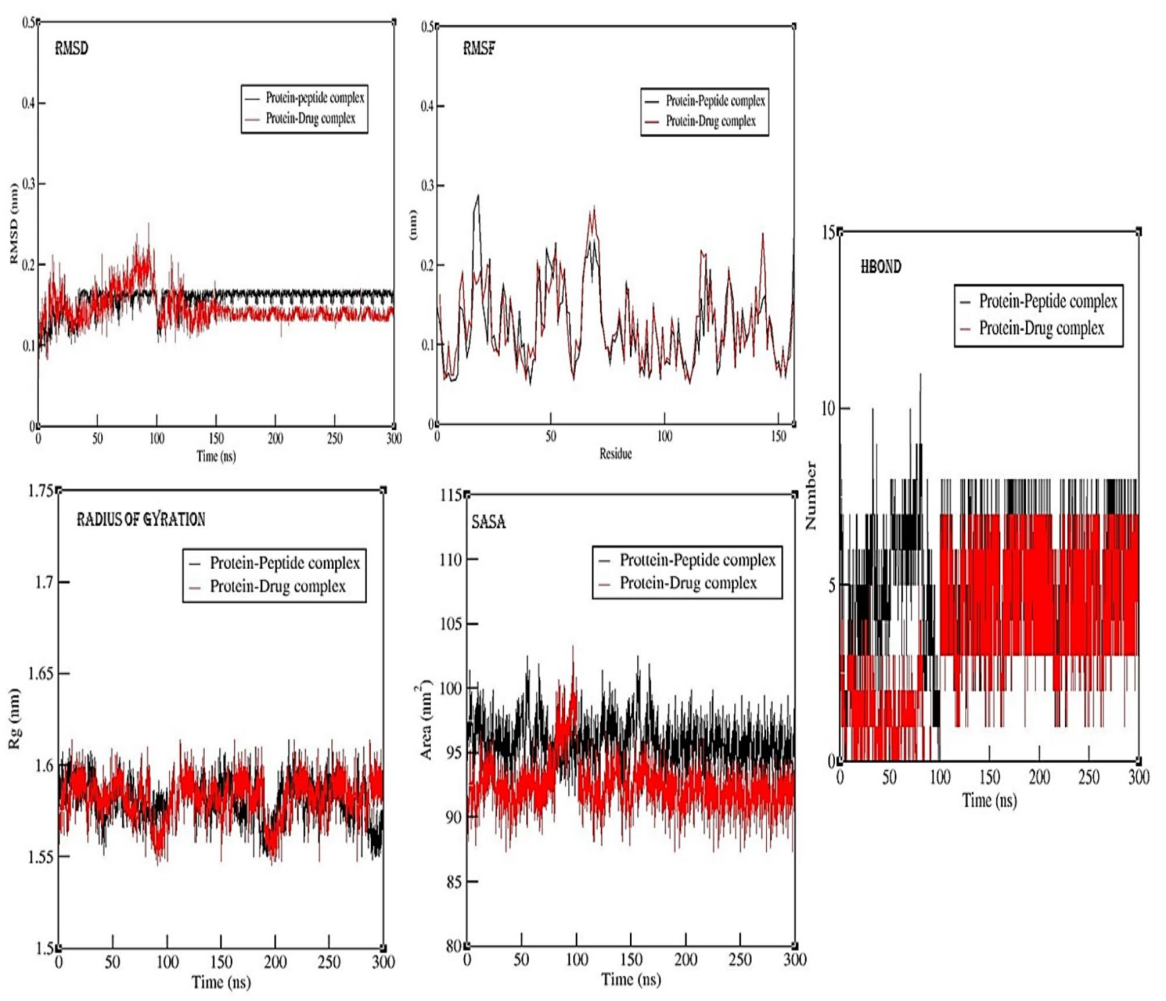

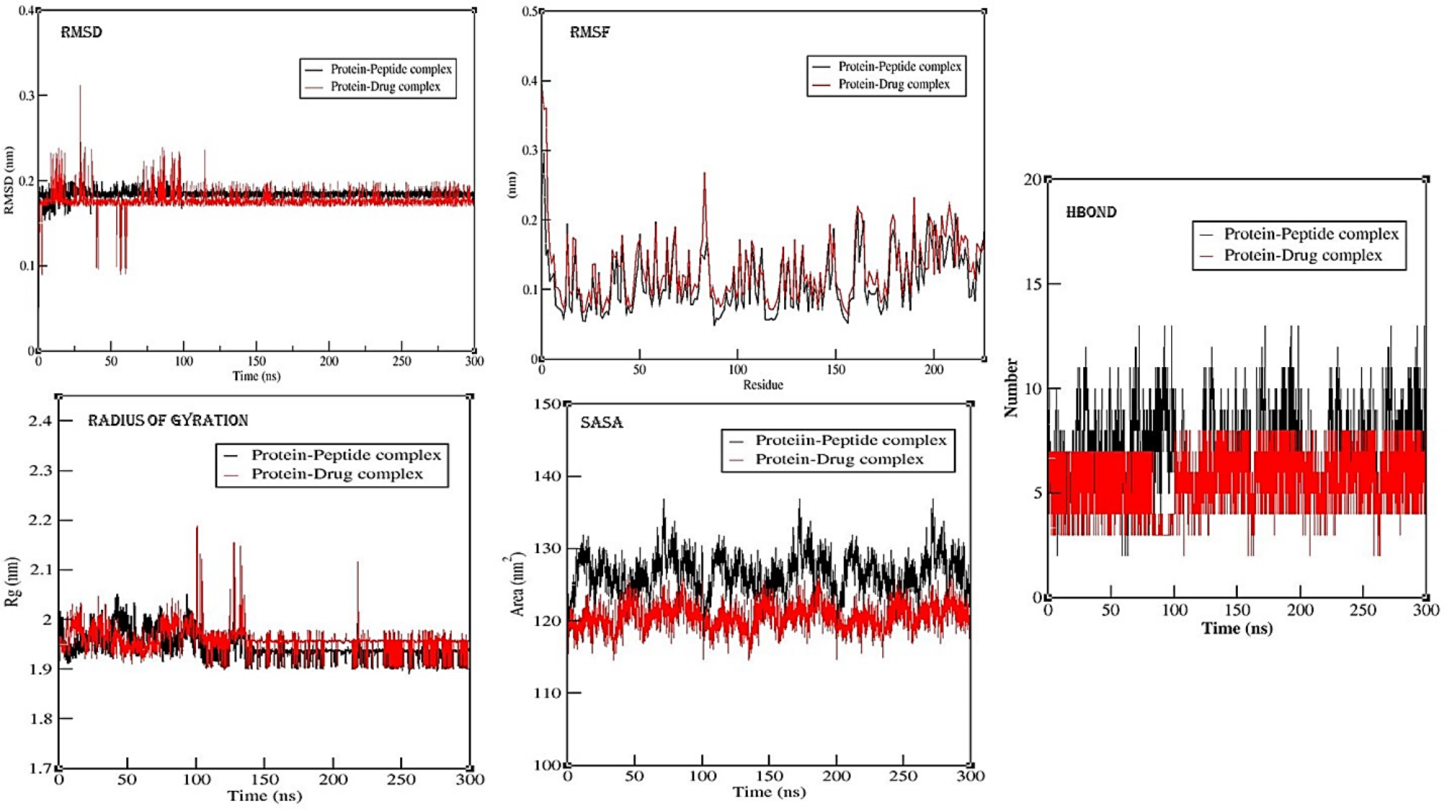
(**A**) Molecular dynamics simulation analysis for the protein-peptide complex for 300 ns using Gromacs-2023.1 version for DHFR enzyme. Results are represented for RMSD - Root Mean Square Deviation, RMSF – Root Mean Square Fluctuations, Rg- Radius of Gyration, SASA- Solvent Accessible Surface Area, and H-Bond – Number of Hydrogen bond formation. (**B**) Molecular dynamics simulation analysis for the protein-peptide complex for 300 ns using Gromacs-2023.1 version for SaTrmK enzyme. Results are represented for RMSD - Root Mean Square Deviation, RMSF – Root Mean Square Fluctuations, Rg- Radius of Gyration, SASA- Solvent Accessible Surface Area, and H-Bond – Number of Hydrogen bond formation.

The RMSD (root mean square deviation) value of protein-peptide complex, for the backbone of the protein during the simulation, there has been a gradual increase from 0.1 nm to 0.15 nm till 22 ns with fluctuations at some time periods. But after 22 ns period, RMSD value was steady for the protein backbone with minor fluctuations at some time frame of simulation. But after 100 ns there has been gradual decrease in the RMSD value of the protein backbone till 140 ns and after that protein backbone RMSD value are steady till 300 ns. When simulation was performed for the protein drug complex, the RMSD value were found to be increasing and reached maximum of 0.24 nm, but when peptide is bound with the protein, the RMSD value has been found to be lower than the protein drug complex for the first 20 ns time period of reaching maximum of 0.157 nm value. This shows that after binding with the peptide, the RMSD fluctuations in the protein has been reduced highlighting the action of peptide over stabilizing the protein backbone. Further, for protein-drug complex, the RMSD value of protein backbone rises till 100 ns and then gradually decreases till 140 ns and after that RMSD value is steady but it is lower than protein-peptide complex. The lower RMSD value of protein backbone for drug complex is might be because of the presence of peptide. RMSF (root mean square fluctuations) for protein-peptide complex, major fluctuations around 16–19, 62–68, 116–120, and 140–144 amino acid residues were observed and RMSF for protein-drug complex, major fluctuations around 16–19, 62–68, 116–120, and 140–144 amino acid residues were observed. The RMSF attributes helps highlight specific modifications within the protein chain. As depicted in the results figures for the protein peptide complex simulation, fluctuations in RMSF values for the amino acids along the protein backbone are more evident at C- and N- terminal in comparison to other regions of the protein. When analysing RMSD, it serves as an indicator of simulation equilibrium; minor fluctuations towards the simulation’s end align with a thermal average structure. For compact, small proteins alterations in the range of 0.1–0.3 nm are within acceptable limits. However, deviations exceeding the range of 0.3 nm are suggesting significant conformational shifts occurring during the simulation or maybe the protein is a membrane protein (Fig. 3A).

Other interactions of the protein-peptide and protein-drug complex, during simulation such as H-bond, radius of gyration and sasa were analysed. From Hbond analysis graph, it has been observed that number of hydrogen bonds is more in protein peptide complex when compared to the protein ligand complex. For protein peptide complex, during 0–30 ns time frame, the number of hydrogen bonds is varied from 2 to 7 number of bonds, highest number of Hbonds 10, & 12 were observed for 35 ns and 80 ns respectively. After 100 ns the number of hydrogen bond formation is uniform for major part of the simulation till 300 ns with maximum number 7–8 number of hydrogen bond being formed. Similarly, for protein-drug complex, number of Hbonds were varied from 0 to 5 over the simulation for 100 ns, but highest number of Hbonds 5 number were observed for 25, & 78 ns respectively. After 100 ns, the number of hydrogen bonds are uniform till 300 ns with the average of 6–7 hydrogen bonds formation. From radius of gyration plot, it has been observed that for both peptide and drug complex, that protein is undergoing some conformational changes upon the binding, but most of the duration of simulation protein was stable and compact. From SASA analysis, it can be inferred that protein is more susceptible to solvent, thereby making the protein unbounding or changes in the conformational states of the protein. Whereas for the protein-drug complex, the solvent accessible is less but at the end of the simulation trajectory there has been increase in the SASA value, thereby inferring the possibility of unfolding of the protein (Fig. 3A).

### Analysis of the peptide against m1A22-tRNA methyltransferase (TrmK) protein complex simulation

The RMSD (root mean square deviation) value for the backbone of the protein-peptide complex during the simulation, there has been a stationary fluctuation increase from 0.18 nm to 0.2 nm till 22 ns with fluctuations at some time periods. But after 22 ns period, RMSD value was steady for the protein backbone with major fluctuations for 42–56 ns time frame of 0.35 nm and minor fluctuations at some time frame of simulation trajectory. After 100 ns, the RMSD value for protein backbone are steady with average value of 0.18–19 nm. This indicates that upon binding of peptide, protein backbone is getting more stable for longer run simulation. When simulation was performed for the protein drug complex, the RMSD value were found to have more fluctuations increasing and reached maximum of 0.32 nm. Whereas after 40 ns the RMSD value is stable for around 70 ns and then there has been a major fluctuation in the RMSD value resulting in the instability of the protein. But when the peptide is bound with the protein, the RMSD value has been found to be stable after the 60 ns and there has been very minor fluctuations rather than having a major fluctuation in the case of protein-drug complex. But for protein-drug complex also, the RMSD value of the protein backbone is steady after 125 ns and the average RMSD value is found to be 0.165–0.17 nm which is lesser than the protein-peptide complex because of the presence of peptide which might be influencing the RMSD value of the protein backbone in its presence of binding. This shows that after binding with the peptide, the RMSD fluctuations in the protein has been reduced highlighting the action of peptide over stabilizing the protein backbone. RMSF (root mean square fluctuations) for protein-peptide and protein –drug complex, major fluctuations were at the start of the protein residue, and then around 76–80, and 140–170 amino acid residues were observed. The RMSF attributes helps highlight specific modifications within the protein chain. As depicted in the results figures for the complex simulation, fluctuations in RMSF values for the amino acids along the protein backbone are more evident at C- and N- terminal in comparison to other regions of the protein. When analysing RMSD, it serves as an indicator of simulation equilibrium; minor fluctuations towards the simulation’s end align with a thermal average structure. For compact, small proteins alterations in the range of 0.1–0.3 nm are within acceptable limits. However, deviations exceeding the range of 0.3 nm are suggesting significant conformational shifts occurring during the simulation or maybe the protein is a membrane protein (Fig. 3B).

Other interactions of the protein-peptide and protein-drug complex, during simulation such as H-bond, radius of gyration and SASA were analysed. From Hbond analysis graph, it has been observed that number of hydrogen bonds is more in protein peptide complex when compared to the protein drug complex. For protein peptide complex, the maximum number of hydrogen bonds formation is 12–13 and the average number of HBond is 10 number. Whereas, for protein-drug complex the maximum number of hydrogen bond formation is average of 8–9 number. From radius of gyration plot, it has been observed that for both peptide and drug complex, that protein is undergoing some conformational changes upon the binding. After 140 ns the gyration of the protein is being found to be steady till 300 ns indicating that the protein is not undergoing major conformational changes in the structural form for both protein-peptide and protein-drug complex. Similarly, from SASA analysis, it can be inferred that protein is more susceptible to solvent, during some time period of simulation, whereas it is uniform for the major part of the simulation of 300 ns time duration for both protein-peptide and protein-drug complex (Fig. 3B).

### Molecular mechanics/Poisson–Boltzmann surface area (MM-PBSA) analysis

MMPBSA analysis were performed to calculate the free energy difference between the bound and unbound states of the complex in different solvation states for the same molecule. For both the complex analysis were performed and the ΔG Energy were calculated. The more negative energy the better the interactions and the stability of the protein-peptide complex during the simulation. MMPBSA results also correlating the docking study and the molecular dynamics simulation results.

**Fig. 4:**
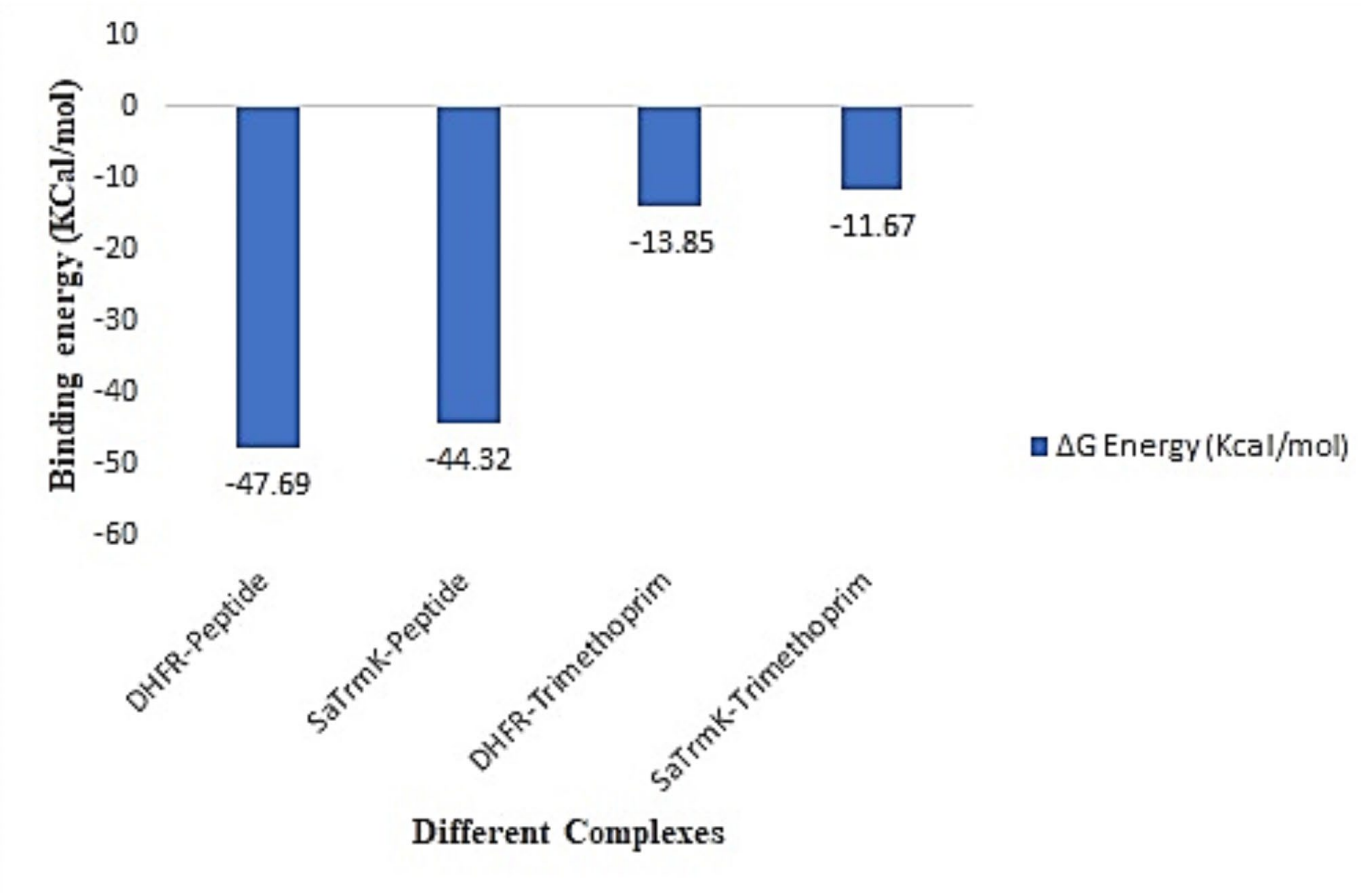
MMPBSA analysis of 300ns trajectory for DHFR and SaTrmK enzyme – peptide and Trimethoprim complex using gmx MMPBSA tool.

From the graph (Fig. 4) it has been observed that delta energy for the complex was found to be -47.69 Kcal/mol. In the case of protein drug complex, the Delta energy for the complex was found to be -13.85 Kcal/mol. Similarly, for *Sa*TrmK protein from the graph it has been observed that Delta energy for the complex was found to be -44.92 Kcal/mol, whereas for the protein-drug complex, the delta energy was found to be -11.67 Kcal/mol. From this it can be inferred that the higher delta energy for the protein peptide complex is stating that the complex is having more affinity in comparison to the protein drug complex. It is also inferred that the due to the presence of peptide, the delta energy is high for the protein peptide complex.

## MIC and MBC determination

To evaluate the antimicrobial activity and specificity of the peptides, MIC was tested against a different strains of *Staphylococcus aureus* (MSSA, MRSA and MDR-SA). From the MIC determination assay, it has found that peptide 2 µg/ml is able to inhibit the growth of the MSSA strain, but for MRSA strain the concentration increases to 4 µg/ml and for multi-drug resistance *S. aureus* strain, it has increased to 8.5 µg/ml. Similarly, for MBC the peptide concentration has not changes for MSSA and MRSA strain, but for MDR strain, the MBC concentration has been increased from 8.5 µg/ml to 10 µg/ml concentration. For comparison, Trimethoprim was used as a standard drug control and MIC for MSSA, MRSA, and MDR-SA strain was found to be 2.5. 5, 9.5 µg/ml; and for MBC concentration has been increased to 4, 7, 12 µg/ml respectively (Table 14).

**Table 14:** The MIC, MBC values of AM1 and Trimethoprim against MSSA, MRSA and MDR-SA strain of *S. Aureus*. The MIC and MBC assays were conducted in triplicate for each strain, and the values are presented as the mean ± standard deviation (SD).

Strains	MIC (µg/ml)		MBC (µg/ml)	
	AM1	Trimethoprim	AM1	Trimethoprim
ATCC 33,591 (MRSA)	4	5	4	7
ATCC BAA-44	8.5	9.5	10	12
ATCC 25,923 (MSSA)	2	2.5	2	4

### Time-kill kinetics assay

**Fig. 5:**
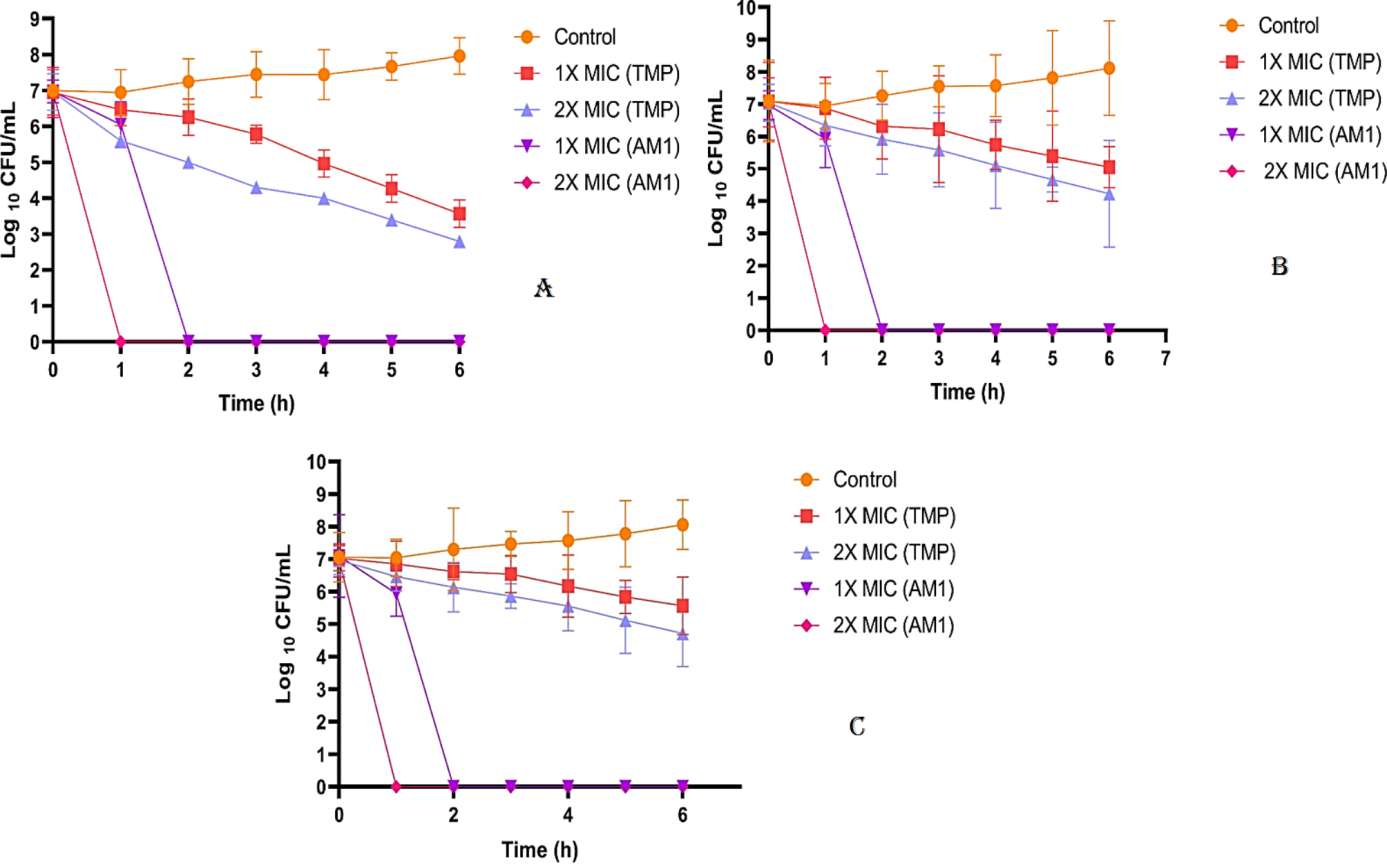
Time-Kill kinetics of AM1 and Trimethoprim at 1X MIC and 2X MIC against MSSA, MRSA and MDR-SA strain of *S. aureus*. (**A**) MSSA, (**B**) MRSA, (**C**) MDR-SA. The assays were performed in triplicate for each bacterial strain, with data presented as mean Log CFU/ml ± standard deviation (SD).

The time-kill kinetics of AM1 peptides at 1 × MIC (2, 4, 8.5 µg/ml) and 2 × MIC (4, 8, 17 µg/ml) against different strains of *S. aureus* (MSSA, MRSA, MDR-SA) were compared with those of Trimethoprim 1X MIC (2.5, 5, 9.5 µg/ml) and 2X MIC (5, 10, 19 µg/ml) concentration respectively. For MSSA strain at 1 × MIC concentration (2 µg/ml) of AM1 peptides, the viable cell count was obtained till 2-hour and after 2-hour no growth is been observed (Fig. 5).

Whereas for 2X MIC (4 µg/ml), AM1 peptide concentration, the growth is inhibited before 1-hour duration, probably after 30–40 min of incubation. Similarly, for MRSA and MDR-SA strain, AM1 peptide no growth was obtained after 1-hour post incubation for 2X MIC concentration. But for TMP, viable cell growth was obtained after 6 hours’ incubation for both MRSA and MDR-SA strain indicating that the effect of TMP over the resistant strains are slow and it takes longer time to completely inhibit the growth. In the MIC assay we obtained the maximum inhibition for the bacteria for both peptides and antibiotic after 18 h of incubation. But in the time-kill assay, MIC of peptides is able to inhibit the growth of bacteria after 2 h, but for antibiotic it is not inhibiting completely in 6 h. In the MIC assay, the effects were observed after 18 h of incubation, whereas the time-kill assay was conducted over a shorter duration of 6 h. As a result, the antibiotic may not exhibit the same efficiency as the peptides within this limited timeframe. This explains why, despite Trimethoprim showing maximum bacterial inhibition at the MIC concentration, it did not demonstrate the same level of effectiveness during the 6-hour time-kill assay. This suggests that Trimethoprim may act as a bacteriostatic agent rather than bactericidal, while the peptide AM1 exhibits bactericidal activity against *S. aureus*.

### Stability assay

**Fig. 6:**
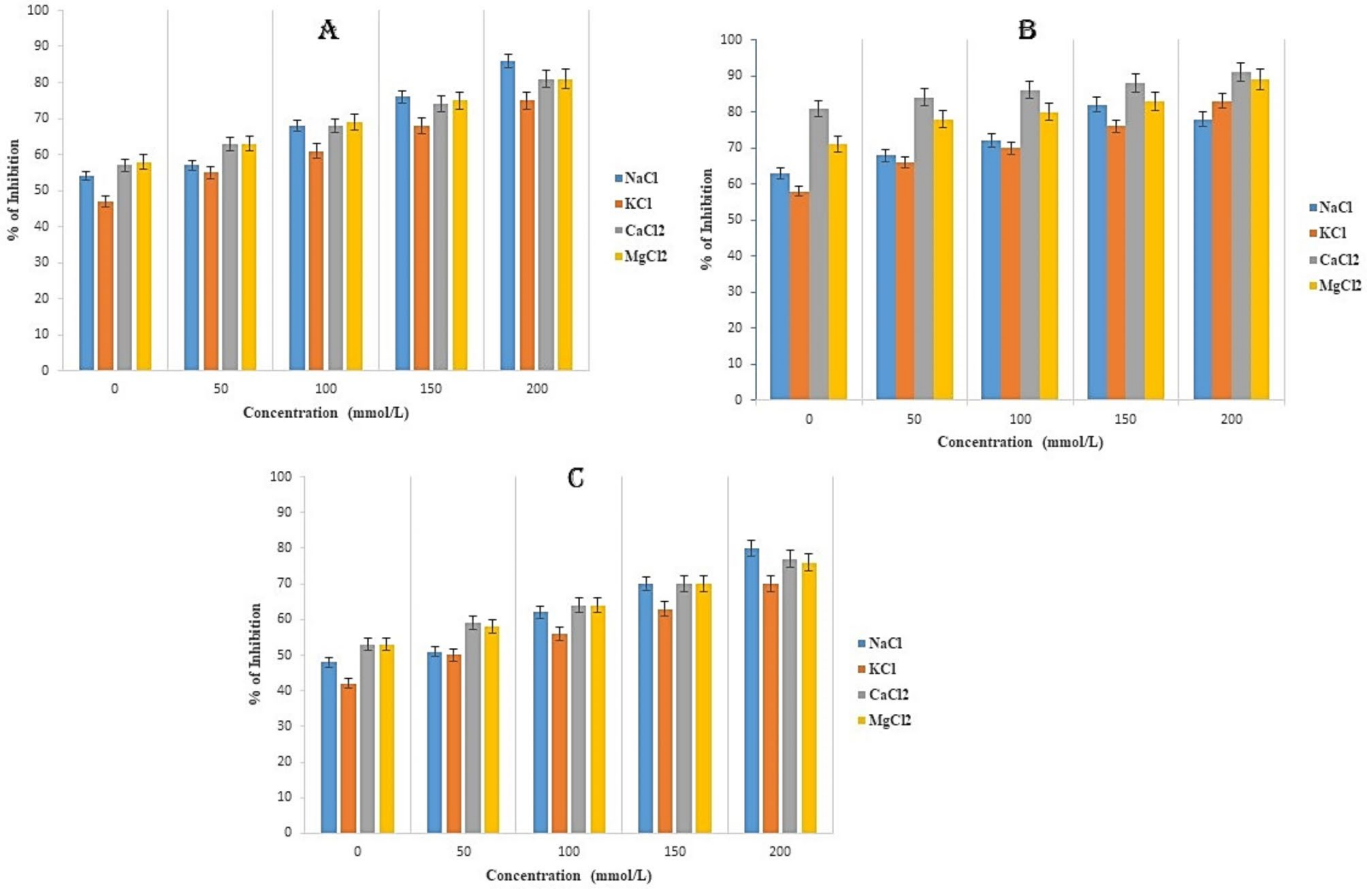

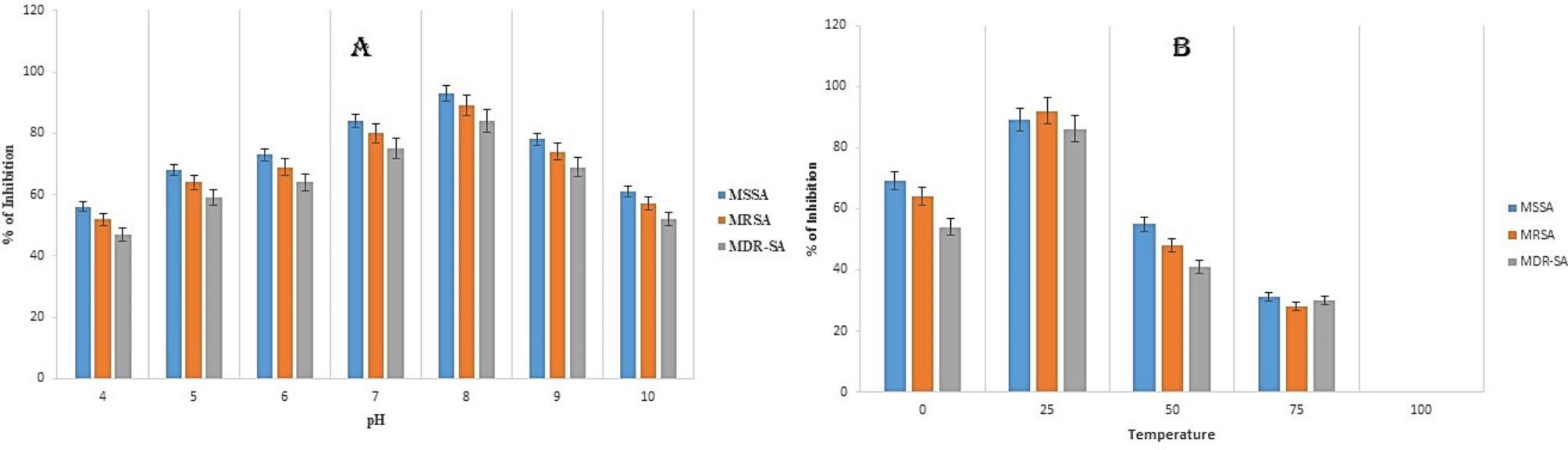
(**a**) Stability assay of AM1 at 1X MIC for different concentration of salt ions ranging from 0 to 200 mmol/L against MSSA, MRSA and MDR-SA strain of *S. aureus*. **A** – MSSA, **B** – MRSA, **C** – MDR-SA. The assays were performed in triplicate for each bacterial strain, with data presented as mean ± standard deviation (SD). (**b**) Effect of different pH (**A**) and temperatures (**B**) of 1X MIC peptide against different strains of *S. aureus.* The assays were performed in triplicate for each bacterial strain, with data presented as mean ± standard deviation (SD).

The stability assay for the peptides were determined using different salt ions, pH and temperature against different strains of *S. aureus* (MSSA, MRSA, and MDR-SA). For the study of the stability effect of peptides, 1X MIC value were taken from the MIC table specific to each strain of S. aureus for their particular antimicrobial effects. From the results, it has been observed that the effect of salt ions alters the efficiency of the peptides against the *S. aureus*, with the increasing concentration of salt ions, the efficacy of the peptides has been also increased and therefore killing the bacterial growth obtained at OD 600 nm. Different salt ions such as NaCl of Na⁺ and Cl⁻ ions can interact with charged residues in the peptide, potentially stabilizing the peptides, MgCl2 of Mg²⁺ ions can stabilize the peptide by interacting with negatively charged residues (e.g., glutamate and aspartate), Similar to NaCl, KCl of K⁺ and Cl⁻ ions can interact with charged residues, influencing the stability of the peptides and CaCl2 of Ca²⁺ ions can strongly interact with negatively charged residues, potentially stabilizing the peptide structure (Fig. 6a). Similarly for the effect of pH, peptide is stable till the pH of 9 and after that the efficiency of the peptides starts decreasing, which has been observed from the results (Fig. 6b). Then for the effect of temperature, the peptide activity has been reduced from the temperature of 50 °C, therefore inferring that the peptide is not heat tolerant beyond the 50 °C.

## Discussions

The rise and swift propagation of methicillin-resistant *S. aureus* strains pose significant challenges for clinical interventions. Reports suggest that patients infected with methicillin-resistant *S. aureus* face heightened clinical risks, particularly an increased mortality risk^37^. Consequently, it is imperative to bolster the prevention of MDR *S. aureus* infection, discover effective antibiotic alternatives, and pinpoint efficacious treatments. Among various novel antibacterial agents, antibacterial peptides, due to their antibacterial activity against a range of pathogens, hold the potential to supplant traditional antibiotics in treating infections caused by MDR strains^38^. While the exact mechanism of action of antimicrobial peptides (AMPs) is not completely understood, it is widely accepted among researchers that AMPs primarily exert their bactericidal effects by creating pores on bacterial surfaces, disrupting membrane structures, and causing extensive leakage of bacterial cell contents, thereby leading to bacterial death. Studies have shown that AMPs can enhance cell membrane permeability, compromise cell membrane integrity, and trigger ion release^39–41^. Furthermore, AMPs have the ability to penetrate cells, bind to DNA, induce bacterial death, and inhibit the formation of biofilms^42^.

In this study, we have predicted the antimicrobial peptide from the proteome sequences of the *Aegle marmelos* against the drug-resistant *S. aureus* target protein utilizing the computational approach. From the study it has been observed that peptide sequence GKEAATKAIKEWGQPKSKITH have Cell penetrating property, non-hemolytic and non-allergenicity property. From ADMET analysis, it has been found that all the peptides are non-toxic and have good bioavailability property. Further, the AMP were evaluated for anti-*Staphylococcus aureus* using docking and dynamics simulation approach. For docking study, two protein target of *S. aureus* were chosen on the basis of literature study. DHFR and TrmK enzyme one which is involved in the synthesis of the purine and pyrimidines and other one is involved in the methylation of the tRNA in the *S. aureus* during infection cycle^45,47^. Dihydrofolate reductase (DHFR) is an enzyme that catalyzes the reduction of dihydrofolate to tetrahydrofolate, a precursor for purine and thymidylate synthesis^43^. Inhibitors of DHFR, such as trimethoprim, have been developed and are clinically used as they effectively inhibit bacterial folic acid synthesis^44^. In the context of *S. aureus*, inhibitors play a crucial role in combating the infection. Moreover, the spread of plasmid-borne resistance enzymes in clinical *S. aureus* isolates is rendering trimethoprim and iclaprim, both inhibitors of DHFR, ineffective. To continue exploiting these targets, compounds that can broadly inhibit these resistance-conferring isoforms are required^45,46^. Whereas, enzyme m1A22-tRNA methyltransferase, also known as TrmK, in *S. aureus*, is tasked with transferring a methyl group from SAM to the N1 of adenine 22 in tRNAs12. This enzyme is vital for the survival of *S. aureus* during an infection. Notably, TrmK does not have a counterpart in mammals, making it a promising target for antibiotic development^47^.

From the docking and molecular dynamics simulation study, it has been observed that the peptide sequences GKEAATKAIKEWGQPKSKITH were able to have maximum binding affinity against both the target protein. From the simulation results, we confirmed that the peptide has been able to stabilize the target protein (DHFR and SaTrmK) in comparison to the standard drug Trimethoprim. RMSD is a measure of the average deviation of the atomic positions from a reference structure over time. It provides insight into the overall stability of the protein structure during the simulation. A lower RMSD value generally indicates that the complex remains close to its initial conformation, suggesting a stable interaction. In our study, proteins with consistently low and stable RMSD values were prioritized, as they demonstrated stable binding of the peptides to the target DHFR and TrmK proteins, which is essential for effective inhibition. RMSF measures the flexibility of individual amino acid residues within the protein throughout the simulation. Regions with high RMSF values indicate greater flexibility, which could correspond to less stable interactions or regions of the proteins that do not contribute to stable binding. We used RMSF to identify proteins that exhibited minimal fluctuations at critical binding sites, ensuring that the selected peptides maintain a firm and stable interaction with the target proteins. Rg reflects the compactness of the proteins structure, indicating how tightly the atoms are packed around the center of mass. A stable Rg value during the simulation suggests that the proteins maintains its compactness, which is often correlated with stability in the binding pocket. Proteins with stable and low Rg values were favored, as they are less likely to undergo significant conformational changes that could disrupt binding. Furthermore, MMPBSA analysis were performed to validate the molecular dynamics simulation for 300 ns trajectory. The Molecular Mechanics Poisson-Boltzmann Surface Area (MMPBSA) method is a widely used approach to estimate the binding free energy of biomolecular complexes. Despite its popularity, MMPBSA has several limitations that must be considered. MMPBSA calculations are heavily dependent on the accuracy of the force field used in the molecular dynamics simulations. Force fields are mathematical models that approximate the interactions between atoms in a molecule. If the force field does not accurately represent the specific interactions in the system, the free energy calculations may be biased or inaccurate. For example, certain force fields might not fully capture the nuances of hydrogen bonding or van der Waals interactions, leading to potential discrepancies in the binding energy estimates. MMPBSA typically uses an implicit solvent model to account for the solvation effects, which approximates the effect of the solvent rather than explicitly simulating solvent molecules. While this approach reduces computational cost, it may not accurately capture the detailed solvent dynamics, especially in systems where specific solvent interactions play a critical role in binding. MMPBSA primarily focuses on the enthalpic contributions to binding free energy, often neglecting or inadequately estimating the entropy changes associated with ligand binding. This simplification can lead to an incomplete understanding of the true binding affinity, particularly in cases where entropy plays a significant role. Beyond these limitations, MMPBSA remains a valuable tool for providing qualitative insights into the relative binding affinities of different peptides, which can guide further experimental validation.

From this result, we can hypothesize that the peptide can interact in bidirectional way of targeting the *S. aureus* virulence pattern. First it can target the nucleotides synthesis and therefore it will inhibit the growth of the bacteria. Other way of targeting can be carried out through TrmK enzyme which is especially present in *S. aureus*, which will the most prominent target in reducing the virulence pattern during infections cycle of *S. aureus*.

Recent research highlights several promising antibacterial agents with activity against multidrug-resistant *Staphylococcus aureus* (MDR S. aureus). These include the photosensitizer S-PS, silver nanoparticles (AgNPs), the antimicrobial peptide sublancin, cell-penetrating peptides (CPPs), and antibiotic combination therapy^48–50^. Among these, antibacterial peptides stand out as potential alternatives to traditional antibiotics for treating MDR infections. In our study, AM1 peptide has been able to inhibit the growth of the *S. aureus* in lesser MIC concentration in comparison to the standard drug trimethoprim. Thus indicating that it can have better effect over the cell membrane disruption of the bacteria. Similar results were also obtained from time kill kinetics, where the 2X MIC of AM1 peptide has been able to completely inhibit the growth of MDR-SA strain after 1-hour which infers that it can acts as a bacteriostatic in treatment of multi-drug resistance *S. aureus* infections.

For instance, the AMP 1,4-naphthoquinone effectively prevents Gram-positive bacterium *Staphylococcus aureus* from forming biofilms by accumulating cellular reactive oxygen species (ROS)^51^. Similarly, AMPNT-6 combats attachment and biofilm formation in the Gram-negative bacterium *Shewanella putrefaciens*^52^. Additionally, the antibacterial peptide Ctn [15–34] exhibits antibiofilm activity against fungi^53^. Notably, the synthetic peptide SHABP reduces oral plaque biofilm formation, offering insights for developing oral anti-biofilm treatments^54^. Overall, AMPs represent a promising avenue for addressing biofilm-related infections and serve as valuable references for other anti-biofilm formulations.

While in silico and in vitro studies provide valuable insights into the potential efficacy of antimicrobial peptides, in vivo evaluation is essential for confirming their therapeutic potential and safety. In vivo studies are critical for understanding the pharmacokinetic properties of peptides, such as absorption, distribution, metabolism, and excretion (ADME), as well as their pharmacodynamic effects, including their interaction with biological targets in a complex living system. These studies can reveal how the peptide is processed by the body and whether it reaches the target site in effective concentrations. Also, to assess the toxicity and safety profile of the peptides, which cannot be fully determined through in vitro or in silico methods. These studies help identify any potential adverse effects that could limit the clinical use of the peptide. The in vivo environment is far more complex than in vitro systems, with factors such as immune response, bioavailability, and interaction with other biological molecules playing significant roles. Testing the peptides in vivo is essential to determine whether the observed in vitro efficacy translates to effective treatment outcomes, particularly in the context of treating infections caused by pathogens like *Staphylococcus aureus*.

## Conclusion

In conclusion, *Staphylococcus aureus*, a highly adaptable pathogen, presents a spectrum of infections ranging from mild to severe, including life-threatening systemic diseases. To our knowledge, this is the first time we have employed a computational approach to forecast and craft novel antimicrobial peptides using the protein sequences of *Aegle marmelos* (Indian Bael fruit). In summary, the findings from this study highlight the potential of the identified peptide sequences to serve as valuable candidates for further in-vivo studies targeted at combating drug-resistant *S. aureus* infections. These peptides hold promise as potential therapeutic agents in the fight against antibiotic-resistant strains, offering new avenues for addressing the growing challenges posed by *S. aureus* infections.

## Electronic supplementary material

Below is the link to the electronic supplementary material.

Supplementary Material 1

## Data Availability

The datasets generated and/or analyzed during this study are available from the corresponding author upon reasonable request.
