# Supplementary material for: In-silico and in-vitro study of novel antimicrobial peptide AM1 from Aegle marmelos against drug-resistant Staphylococcus aureus
Source: Sci Rep. 2024 Oct 28;14:25822. doi: 10.1038/s41598-024-76553-0 (PMC11519352; doi:10.1038/s41598-024-76553-0)
Supplement: Supplementary file 1 — Supplementary Material 1 [file 41598_2024_76553_MOESM1_ESM.docx]

**In-silico and in-vitro study of novel Antimicrobial peptide AM1 from *Aegle marmelos* against drug-resistant *Staphylococcus aureus***

Rudra Awdhesh Kumar Mishra, Gothandam Kodiveri Muthukaliannan**^*^**

School of Biosciences and Technology, Vellore Institute of Technology, Vellore, Tamil Nadu, India

**Corresponding author**

Dr K M Gothandam,

Professor,

School of Biosciences and Technology,

Vellore Institute of Technology,

Vellore – 632014, Tamil Nadu, India

**Email Id:** [gothandam@gmail.com](mailto:gothandam@gmail.com)

**Contact Number:** +91 9489337967

**Table S1 (a). Physicochemical properties for predicted Antimicrobial peptides**

| **Peptide Sequence** | **Peptide Length** | **Peptide mass (Daltons)** | **Charge** | **pI** | **Hydrophobicity (Wimley-White whole-residue)** | **Hydropathy value** | **Boman Index (kcal/mol** |
| --- | --- | --- | --- | --- | --- | --- | --- |
| KAGVKDYK | 8 | 908.07 | +2 | 9.53 | 3.51 | -1.3625 | 2.34 |
| KAGVKDY | 7 | 779.89 | +1 | 8.50 | 2.52 | -1 | 1.88 |
| NLSHYYSGSSK | 11 | 1242.31 | +1/25 | 8.50 | -0.33 | -1.8181 | 2.26 |
| KRLGSE | 6 | 688.78 | +1 | 8.75 | 3.4 | -1.55 | 4.13 |
| SVKRLITRM | 9 | 1103.39 | +3 | 12.01 | 1.85 | 0 | 2.79 |
| SCVKSL | 6 | 635.78 | +1 | 7.94 | 0.52 | 0.8333 | 0.35 |
| VRICRNL | 7 | 873.08 | +2 | 10.36 | 1 | 0.357 | 3.04 |
| HVSSQPGRVQLNHLY | 15 | 1734.94 | +1.5 | 8.76 | 1.53 | 0.686 | 2 |
| VRICRNLSHY | 10 | 1260.48 | +2.25 | 9.50 | 0.36 | 0.28 | 2.95 |
| DNKSSSLSVKRLITRMY | 17 | 1998.33 | +3 | 10.28 | 3.38 | 0.636 | 3.02 |
| HIHRAMHAVIDRQKNHGMH | 19 | 2288.64 | +3.25 | 10.84 | 5.03 | -1.010 | 2.97 |
| AGTVVGKL | 8 | 743.90 | +1 | 8.80 | 0.9 | 1.075 | 1.07 |
| KKIRRLGAL | 9 | 1054.35 | +4 | 12.02 | 2.35 | 0.366 | 2.6 |
| KKIRRL | 6 | 813.06 | +4 | 12.02 | 2.73 | -1.416 | 5.18 |
| MPNIYNALVVK | 11 | 1261.55 | +1 | 8.34 | 0.55 | 0.6 | 0.28 |
| TTSPIHK | 7 | 782.89 | +1.25 | 8.44 | 1.71 | 0.914 | 1.97 |
| INNIAKAH | 8 | 880.01 | +1.25 | 8.76 | 1.72 | -0.1875 | 1.25 |
| RRGGKIGLF | 9 | 1003.22 | +3 | 12.01 | 0.64 | -0.333 | 2.19 |
| ETAQRVKQTLQRY | 13 | 1620.83 | +2 | 9.99 | 5.39 | -1.523 | 4.1 |
| FMRWRDRF | 8 | 1213.43 | +2 | 11.70 | -0.68 | -1.3 | 5.35 |
| GLIVSSRKDSLQHF | 14 | 1586.81 | +1.25 | 8.75 | 1.69 | -0.164 | 1.91 |
| LCNSVGR | 7 | 747.87 | +1 | 8.25 | 0.64 | 0.182 | 1.96 |
| KEGVRRLVVV | 10 | 1154.42 | +2 | 10.84 | 4.36 | 0.38 | 2.01 |
| CNSVGRF | 7 | 781.88 | +1 | 8.25 | 0.07 | 0.04 | 2.24 |
| KEGVRRL | 7 | 857.02 | +2 | 10.84 | 4.15 | -1.257 | 4.61 |
| WARWAHKALWHASL | 14 | 1733.01 | +2.5 | 11.00 | -3.72 | -0.25 | 0.65 |
| AHKALW | 6 | 724.86 | +1.25 | 8.80 | -0.91 | -0.1 | -0.11 |
| LNSLLVR | 7 | 814.00 | +1 | 9.75 | -0.25 | 0.971 | 0.87 |
| FCNALGHPISK | 11 | 1186.39 | +1.25 | 8.23 | 0.1 | 0.181 | 0.3 |
| KDPFMHYVRYQGKSIL | 16 | 1982.33 | +2.25 | 9.53 | 1.32 | -0.668 | 1.77 |
| CNALGHPISKSTW | 13 | 1413.61 | +1.25 | 8.23 | -0.35 | -0.24 | 0.77 |
| WYRPLK | 6 | 862.04 | +2 | 9.99 | -1.1 | -1.4 | 2.22 |
| RKVVTERPAH | 10 | 1192.39 | +2.25 | 10.84 | 5.7 | -1.17 | 3.95 |
| IVGKSW | 6 | 688.82 | +1 | 8.75 | -0.96 | 0.45 | -0.54 |
| KASRHVMSE | 9 | 1044.19 | +1.25 | 8.75 | 4.26 | -0.977 | 3.39 |

**Table S1 (b). Instability index and the half-life time for the predicted antimicrobial peptides**

| **Peptide Sequence** | **Instability Index** | **Half Life Time** | | |
| --- | --- | --- | --- | --- |
|  |  | **Mammalian reticulocytes (in-vitro)** | **Yeast (in-vivo)** | **Escherichia coli (in-vivo)** |
| KAGVKDYK | 5.15 - Stable | 1.3 hours | 3 min | 3 min |
| KAGVKDY | 4.46 - Stable | 1.3 hours | 3 min | 3 min |
| NLSHYYSGSSK | 77.76 - Unstable | 1.4 hours | 3 min | >10 hours |
| KRLGSE | 94.77 - Unstable | 1.3 hours | 3 min | 3 min |
| SVKRLITRM | 41.91 - Unstable | 1.9 hours | >20 hours | >10 hours |
| SCVKSL | 45.30 - Unstable | 1.9 hours | >20 hours | >10 hours |
| VRICRNL | 26.20 - Stable | 100 hours | >20 hours | >10 hours |
| HVSSQPGRVQLNHLY | 47.85 - Unstable | 3.5 hours | 10 min | >10 hours |
| VRICRNLSHY | 65.28 - Unstable | 100 hours | >20 hours | >10 hours |
| DNKSSSLSVKRLITRMY | 77.41 - Unstable | 1.1 hours | 3 min | >10 hours |
| HIHRAMHAVIDRQKNHGMH | 100.10 - Unstable | 3.5 hours | 10 min | >10 hours |
| AGTVVGKL | -33.70 - Stable | 4.4 hours | >20 hours | >10 hours |
| KKIRRLGAL | 53.67 - Unstable | 1.3 hours | 3 min | 3 min |
| KKIRRL | 89.65 - Unstable | 1.3 hours | 3 min | 3 min |
| MPNIYNALVVK | 86.36 - Unstable | 30 hours | >20 hours | >10 hours |
| TTSPIHK | 122.80 - Unstable | 7.2 hours | >20 hours | >10 hours |
| INNIAKAH | 53.06 - Unstable | 20 hours | 30 min | >20 hours |
| RRGGKIGLF | 57.94 - Unstable | 1 hours | 2 min | 2 min |
| ETAQRVKQTLQRY | 44.51 - Unstable | 1 hours | 30 min | >10 hours |
| FMRWRDRF | 61.50 - Unstable | 1.1 hours | 3 min | 2 min |
| GLIVSSRKDSLQHF | 60.37 - Unstable | 30 hours | >20 hours | >10 hours |
| LCNSVGR | -3.56 - Stable | 5.5 hours | 3 min | 2 min |
| KEGVRRLVVV | 66.28 - Unstable | 1.3 hours | 3 min | 3 min |
| CNSVGRF | -3.56 - Stable | 1.2 hours | >20 hours | >10 hours |
| KEGVRRL | 90.40 - Unstable | 1.3 hours | 3 min | 3 min |
| WARWAHKALWHASL | 73.41 - Unstable | 2.8 hours | 3 min | 2 min |
| AHKALW | 73.12 - Unstable | 4.4 hours | >20 hours | >10 hours |
| LNSLLVR | 8.57 - Stable | 5.5 hours | 3 min | 2 min |
| FCNALGHPISK | 6.47 - Stable | 1.1 hours | 3 min | 2 min |
| KDPFMHYVRYQGKSIL | 86.69 - Unstable | 1.3 hours | 3 min | 3 min |
| CNALGHPISKSTW | -4.55 - Stable | 1.2 hours | >20 hours | >10 hours |
| WYRPLK | -1.90 - Stable | 2.8 hours | 3 min | 2 min |
| RKVVTERPAH | 41.31 - Unstable | 1 hours | 2 min | 2 min |
| IVGKSW | -34.12 - Stable | 20 hours | 30 min | >10 hours |
| KASRHVMSE | 121.91 - Unstable | 1.3 hours | 3 min | 3 min |

Colony Forming Units (CFU) images for the effect of peptides against different strains of *S. aureus* for MBC, the concentration at which no visible growth has been observed in the spread plates.


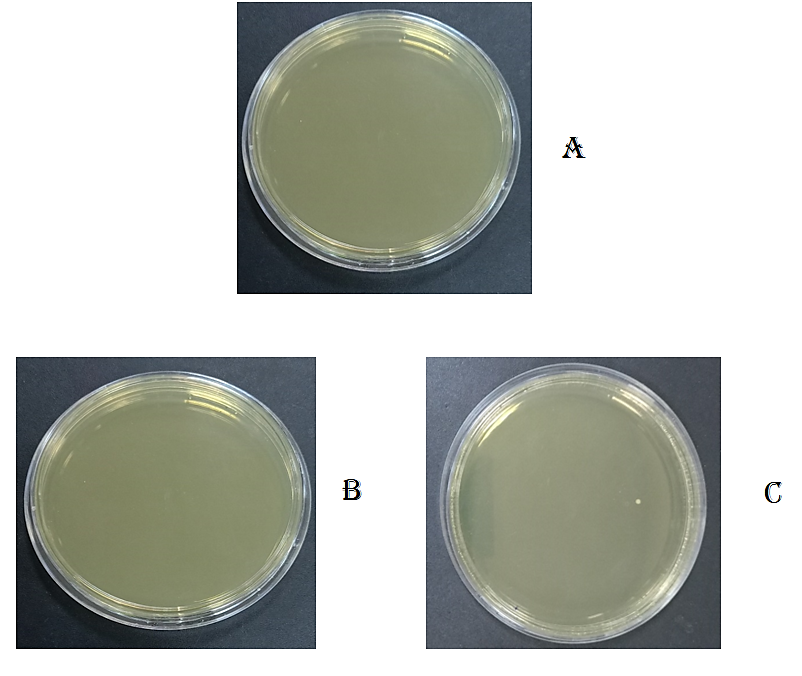


**Figure S1.** Minimum Bactericidal Concentration (MBC) at which no visible growth has been observed on the agar plate after 24 hours of incubation. A – MSSA, B – MRSA, C – MDR-SA
